# Functional characterization and membrane localization of the styrene oxide isomerase from *Rhodococcus opacus* 1CP and *Zavarzinia compransoris* Z-1155

**DOI:** 10.1128/spectrum.01526-25

**Published:** 2025-09-12

**Authors:** Selvapravin Kumaran, Shanice Olanipekun, Latife Sönmez, Lars Janzen, Peter-Leon Hagedoorn, Dirk Tischler

**Affiliations:** 1 Microbial Biotechnology, Ruhr University Bochum 9142, Bochum, Germany; 2 Department of Biotechnology, Delft University of Technology 201231, Delft, the Netherlands; Reichman University, Herzeliya, Israel

**Keywords:** Integral membrane protein, fluorescence microscopy, site-directed mutagenesis, EPR, sfGFP/SUMO, cytosolic termini, membrane anchor, terminal extension, truncation

## Abstract

Styrene oxide isomerase (SOI) is a part of the styrene degradation enzyme complex, performing the isomerization of toxic intermediate styrene oxide into phenylacetaldehyde. For many years, the enzyme was believed to be cofactor-independent, and hence, the mechanism of this enzyme was proposed to be acid-base catalysis. Recently, the presence of heme was identified and reported in SOI from *Pseudomonas* sp. VLB120. Alongside, the membrane localization was also postulated since its discovery but lacks experimental proof. In this study, we highlight the localization of SOIs from two bacterial strains, *Rhodococcus opacus* 1CP and *Zavarzinia compransoris* Z-1155, heterologously overproduced in the cell membrane of *E. coli via* sfGFP-tagged fusions. In addition, the site-directed mutagenesis of acidic and basic amino acids in SOI from 1CP also showcased that histidine-57 is the axial ligand to the heme. Electron paramagnetic resonance (EPR) and biocatalytic assays showed arginine-111 possibly coordinating the propionate group of heme. The functional assays of differently tagged sfGFP with and without linkers, and the truncation of the terminal extension of SOI from 1CP and Z-1155, indicate their possible role in proper substrate channeling. It also supports the previously proposed SOI role as a membrane anchor for other enzymes in styrene degradation pathway.

**IMPORTANCE:**

Styrene oxide isomerase (SOI) catalyzes the isomerization of styrene oxide into phenylacetaldehyde in the side chain oxygenation of the styrene degradation pathway. Despite performing a key role in this pathway, the biology and biochemistry of this enzyme are poorly understood, particularly its catalytic mechanism, membrane localization, and structure-function relationships. SOIs from *Rhodococcus opacus* and *Zavarzinia compransoris* were produced as SUMO/sfGFP/mCherry fusion proteins. We successfully achieved the overproduction and performed site-directed mutagenesis to understand the catalytic mechanism, performed whole-cell assays, used fluorescent microscopy to assess the membrane localization, and constructed terminal truncations to assess structure-function relationships. The site-directed mutagenesis revealed histidine-57 as the axial ligand for heme. The fluorescence microscopy of sfGFP-fusion showed that SOI is a membrane-bound protein with both termini localized in the cytosol. The difference in activity of differently tagged SOI and truncation of the terminal extension showed that the termini might facilitate proper substrate channelling.

## INTRODUCTION

Styrene is one of the most produced compounds in the chemical industry, reaching millions of tons (1). It is a natural component in tar and can be found in plants and food as a volatile and oily substance (2). It is a very important starting material for the synthesis of synthetic polymers such as polystyrene or styrene-butadiene rubber in the petrochemical industry (1, 3). In addition to chemical production, styrene is also formed in nature by plants and microorganisms. Some microorganisms are also able to break it down. Despite a significant amount of release, the ecosystem was not recorded for styrene pollution due to naturally available styrene degradation routes. Numerous microorganisms are capable of using styrene as a sole carbon source. Particularly, styrene was reported to be degraded not only by several *Pseudomonas* strains (4–8) but also by *Xanthobacter* sp. strain 124X (9), *Corynebacterium* sp. AC-5 (10), *Sphingopyxis fribergensis* Kp5.2 (11), *Rhodococcus* sp. ST-5 and ST-10 (12), as well as *Rhodococcus opacus* 1CP (13).

The side chain oxygenation is a well-studied pathway for styrene degradation. Here, styrene is converted to phenylacetic acid with styrene oxide and phenylacetaldehyde as intermediate compounds. First, a two-component styrene monooxygenase (SMO) converts styrene to styrene oxide. The styrene oxide is then isomerized to phenylacetaldehyde by styrene oxide isomerase (SOI), which is then converted into phenylacetic acid by phenylacetaldehyde dehydrogenase (PAD) (8).

SOI is the least understood enzyme in this pathway, where its catalytic mechanism was hypothesized to be acid-base catalysis, following a rare Meinwald rearrangement (14). The reaction mechanism is to isomerize styrene oxide *via* a 1,2 intramolecular hydride shift (15). Recent studies on the overproduction of SOI using SUMO fusion (16) and our collaborative work on cryo-EM structure showcased the presence of heme as a catalytic cofactor (17).

**Fig 1:**
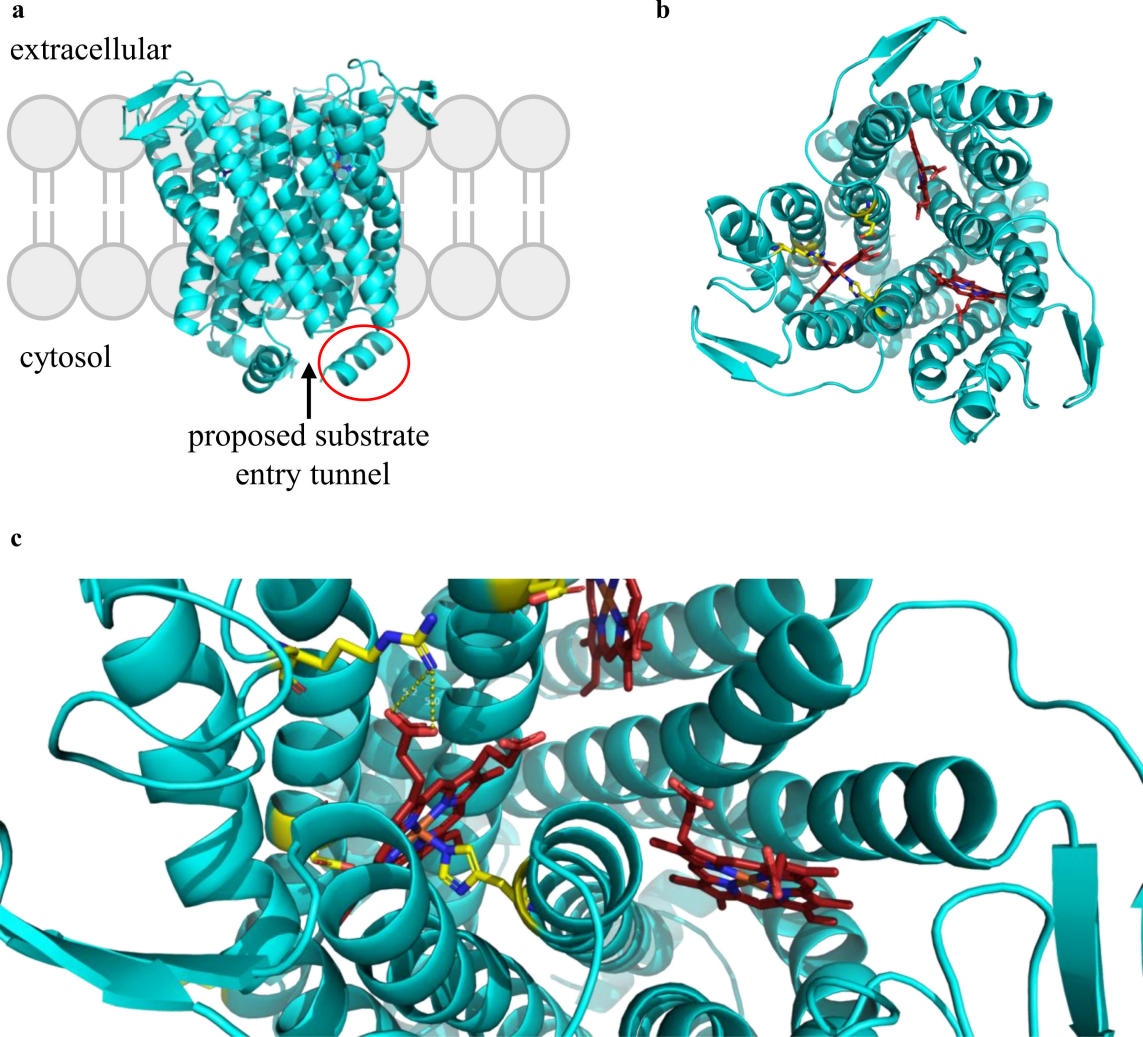
Alphafold3 structure of *Ro*SOI1. (**a**) Trimeric SOI localized in the membrane with both *N-* and *C-*termini localized cytosolically. The substrate tunnel proposed is shown with an arrow. The terminal extension is highlighted in a red circle. (**b**) View from the extracellular side, showing the heme localized at the protomeric interface. The residues chosen for site-directed mutagenesis are given in yellow and heme in red. (**c**) Closer view of active site heme with axial ligand histidine-57. The coordination of arginine-111 with heme’s propionate group is represented in dotted lines.

In order to address the fundamental questions like understanding the catalytic mechanism, the structure-function relationships, and the membrane localization, we employed a holistic approach on the following two candidates that possess *N-*/*C-* terminal extension: SOI from *Rhodococcus opacus* 1CP (*Ro*SOI1) and *Zavarzinia compransoris* Z-1155 (*Zc*SOI). First, we studied the fusion-based approach with SUMO, sfGFP, and mCherry to improve overproduction and enable fluorescence microscopic investigations. These constructs enabled the whole-cell biocatalysis, site-directed mutagenesis, and spectroscopic characterization to identify key catalytic residues. Additionally, we utilized fluorescence microscopy to elucidate the membrane localization and topological orientation of SOI. The altered activity profile depending on the tag position highlighted the impact of terminal extension in *Ro*SOI1 (Fig. 1). In addition, we also generated various terminally truncated variants and examined the effect on activity, localization, and cofactor incorporation.

The current study provides new insights into the functional plasticity of the terminal extension for effective enzyme activity, thereby not only contributing toward a deeper understanding of SOI’s biology but also opening new avenues for engineering the enzyme for industrial applications.

## RESULTS

### Alphafold3-based SOI model prediction

The alphafold3-generated *Ro*SOI1 model (18) with heme as a ligand showed a confidence score of (90 > plDDT > 70) across the transmembrane domain. A low confidence score was recorded near the termini and loops (70 > plDDT > 50), possibly indicating that those regions might be flexible. Cα-superimposition of *Ro*SOI1 model with cryo-EM structure of *Pt*SOI (PDB ID: 8PNV) showed an r.m.s.d value of 0.65 over 152 residues. This strongly supports the identity and integrity of the *Ro*SOI1 model (Fig. 1) produced.

### Site-directed mutagenesis of a highly conserved charged amino acid showed histidine-57 as an axial ligand for heme

Most of the work conducted on SOI was limited to whole-cell or crude extract samples due to its difficulties in solubilization. Until only recently, SOI was successfully purified as a fusion with the SUMO protein solubilized in the Dodecyl-β-D-maltoside (DDM) detergent (16).

**Fig 2:**
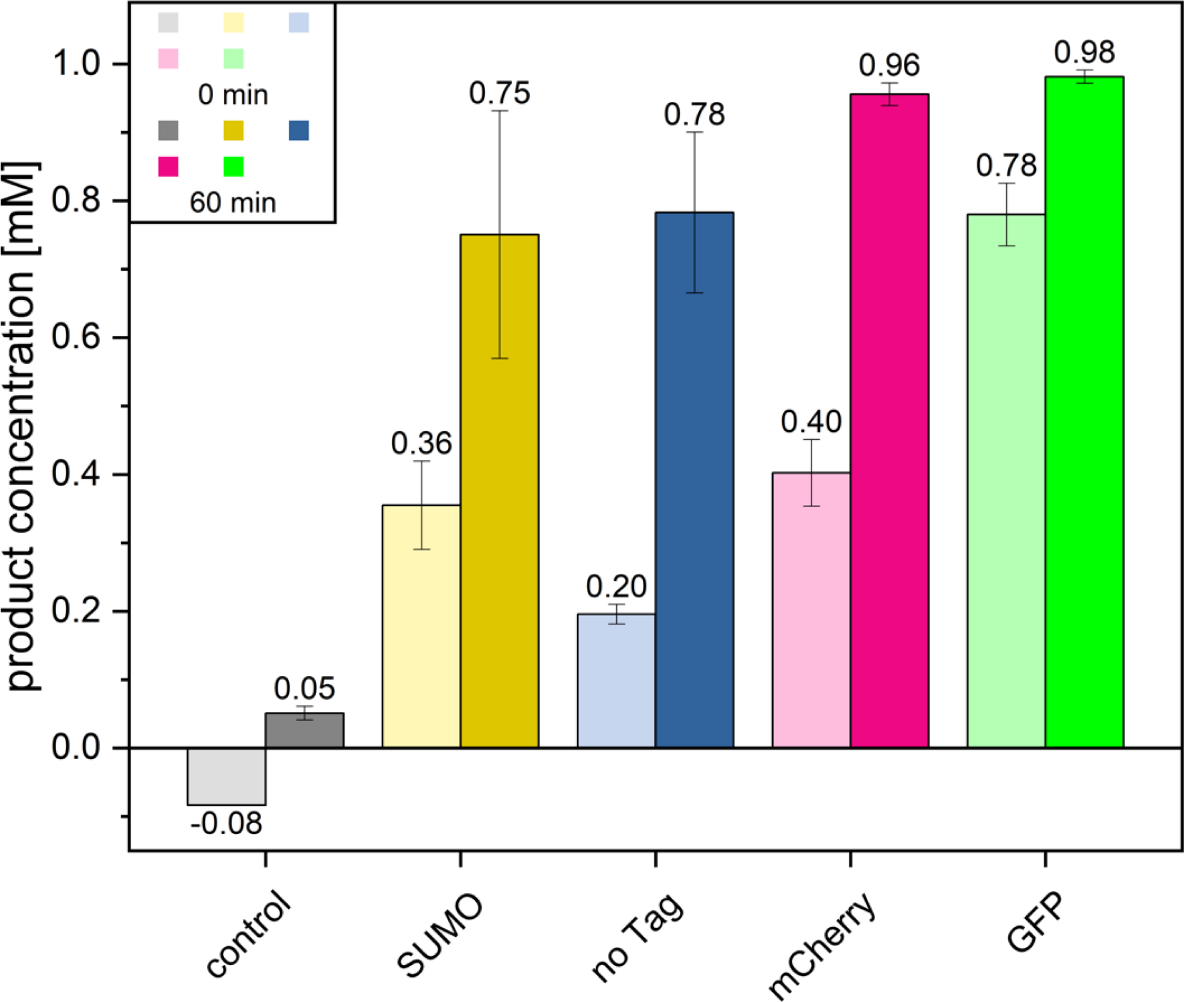
Whole-cell biocatalysis of SOI-fusion proteins. The bar indicates the amount of product 2-indanone produced by differently tagged *Ro*SOI1, along with WT (no tags). The samples were collected and measured in RP-HPLC immediately after adding substrate (0 min -light bars) and after 60 min of biocatalysis (dark bars). All the fusion proteins showed isomerization activity. The assay was performed in triplicate, and the bar indicates the average of the triplicate measurements, and the error bar indicates the standard deviation. The negative control was treated the same, without cells.

To study the fusion-based expression strategies, three *N-*terminal fusion constructs, namely, *SUMO*, *sfGFP*, and *mCherry,* were generated with the *RoSOI1* gene. Instead of the native substrate styrene oxide, the alternative substrate indene oxide was used to test the whole-cell biocatalysis assay. This is because the host *E. coli* contains endogenous aldehyde dehydrogenases that can metabolize or convert the natural product phenylacetaldehyde, whereas the indene oxide converted into 2-indanone, catalyzed by SOI, cannot be metabolized by the host. The whole-cell biocatalysis confirmed that all three fusion proteins retained catalytic activity (Fig. 2). Although GFP and mCherry fused SOI showed relatively higher product concentration in the whole-cell assay, the SUMO-tagged SOI was chosen for site-directed mutagenesis. This selection was based on a previous report that the SUMO-tag enhances the gene expression and protein yield for SOI (16), which would be beneficial for subsequent protein purification and to study the catalytic mechanism.

**Fig 3:**
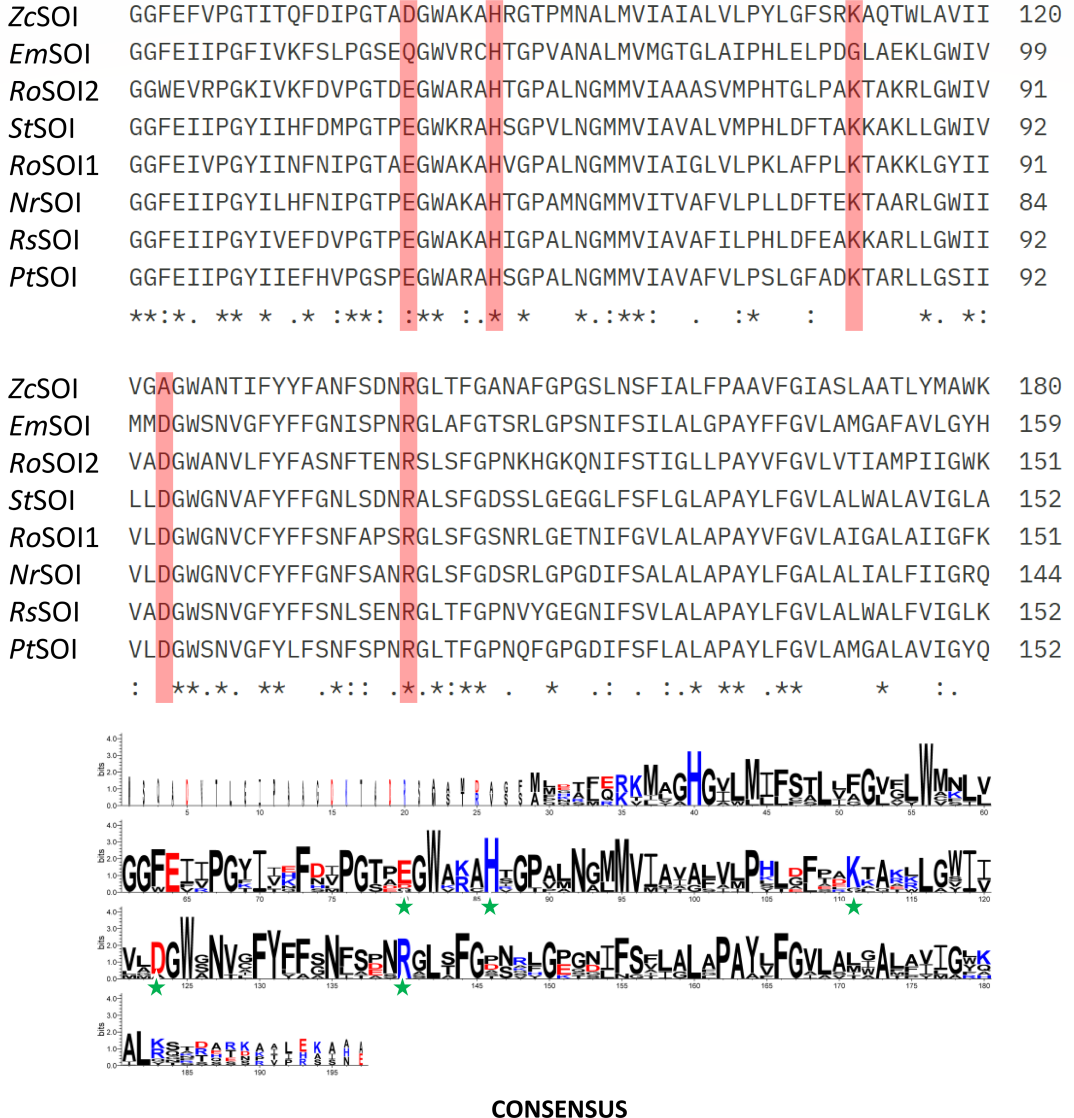
Highly conserved amino acid residues in SOIs. WebLogo 3 shows the amino acids that are conserved in SOI sequences for the prediction of residues relevant to acid-base catalysis. Such residues chosen for site-directed mutagenesis from the model enzyme *Ro*SOI1, E51, H57, K82, D94, and R111 are highlighted with red boxes in the alignment and with a green star in the consensus sequence logo. The alignment was performed with the following SOIs: *Zc*SOI (TDP40089.1), *Em*SOI (KIV94526.1), *Ro*SOI2 (AII82580.1), *St*SOI (WP 022958324.1), *Ro*SOI1 (AII82581.1), *Nr*SOI (WP 028475659.1), *Rs*SOI (MAI33794.1), and *Pt*SOI (AAC23720.1) (the alignment is displayed partially; for a complete Clustal alignment, refer Fig. S8).

**Fig 4:**
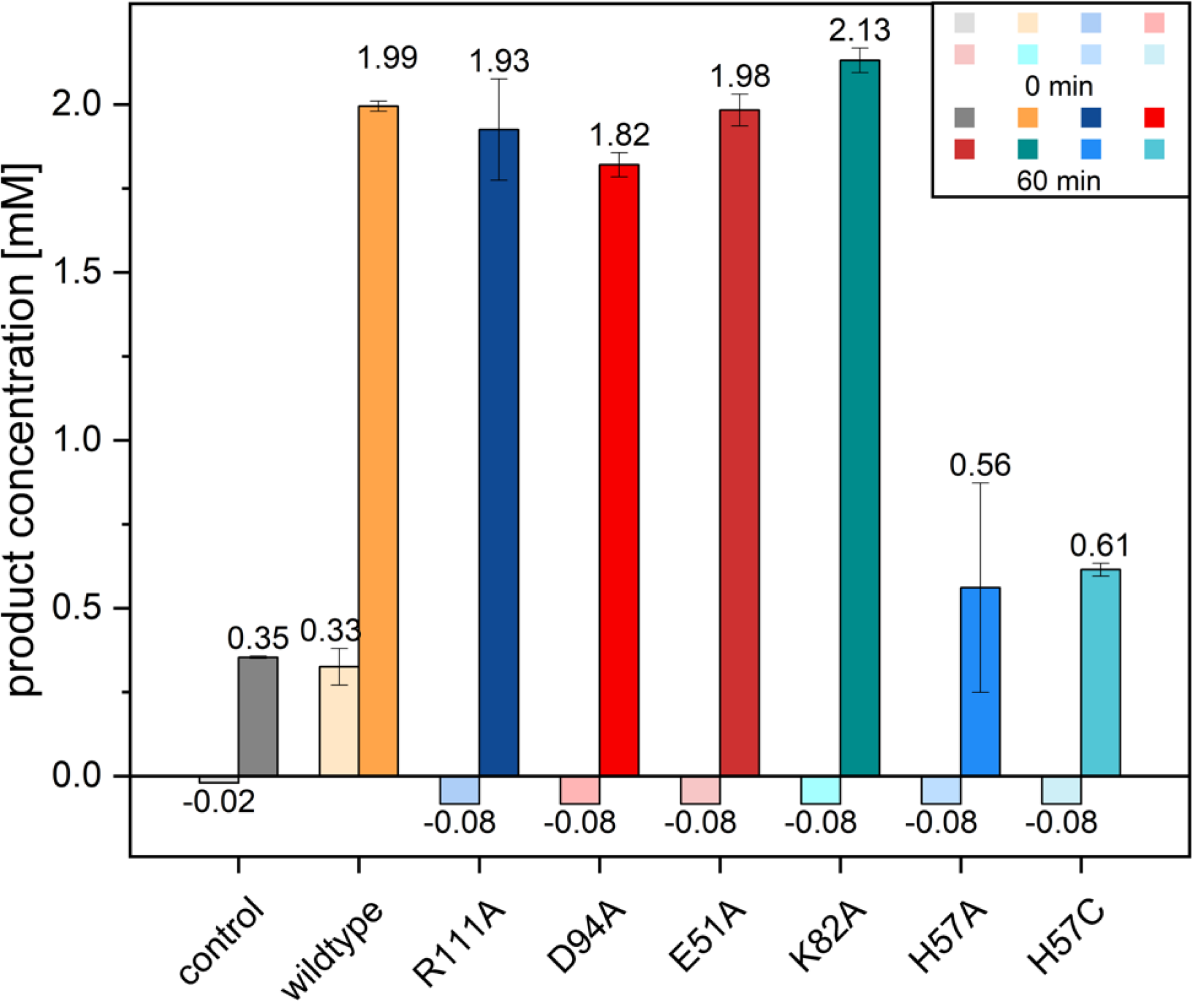
Whole-cell biocatalysis of *Ro*SOI1 variants. The bar indicates the amount of product 2-indanone produced by different variants tagged with *N-*terminal SUMO to *Ro*SOI1. The samples were collected and measured in RP-HPLC immediately after adding substrate (0 min -light bars) and after 60 min of biocatalysis (darker bars). All the variants showed isomerization activity except H57A and H57C. The assay was performed in triplicate, and the bar indicates the average of triplicate measurements, and the error bar indicates the standard deviation. The negative control was treated the same, without cells.

In order to identify key residues in the active site, highly conserved acidic and basic amino acids were given importance. Based on the multiple sequence alignments, the residues E51, H57, K82, D94, and R111 from *Ro*SOI1 were chosen for site-directed mutagenesis and were one-by-one substituted by alanine (Fig. 3). All variants retained activity in the whole-cell biocatalysis assay except H57A, which completely lost the isomerization activity (Fig. 4). Interestingly, purified SOI appeared in reddish-brown color, indicative of presence of heme, which was consistent with the previously reported SOI (17). These findings marked a shift in understanding SOI’s mechanism from acid-base catalysis to a heme-mediated Lewis-acid catalysis (17).

Western blotting and heme staining of SDS-PAGE confirmed the expression, overproduction of fusion proteins, and heme loading in all the constructs. On the contrary, the exchange of histidine to alanine (H57A) and cysteine (H57C, another most common ligand that coordinates heme) lost both activity and heme (Fig. 4; Fig. S1 to S4). This highlights the essential role of H57 as the axial ligand for heme. Notably, the substitution of H57 with cysteine also failed to restore heme binding or activity, emphasizing the histidine-heme coordination.

**Fig 5:**
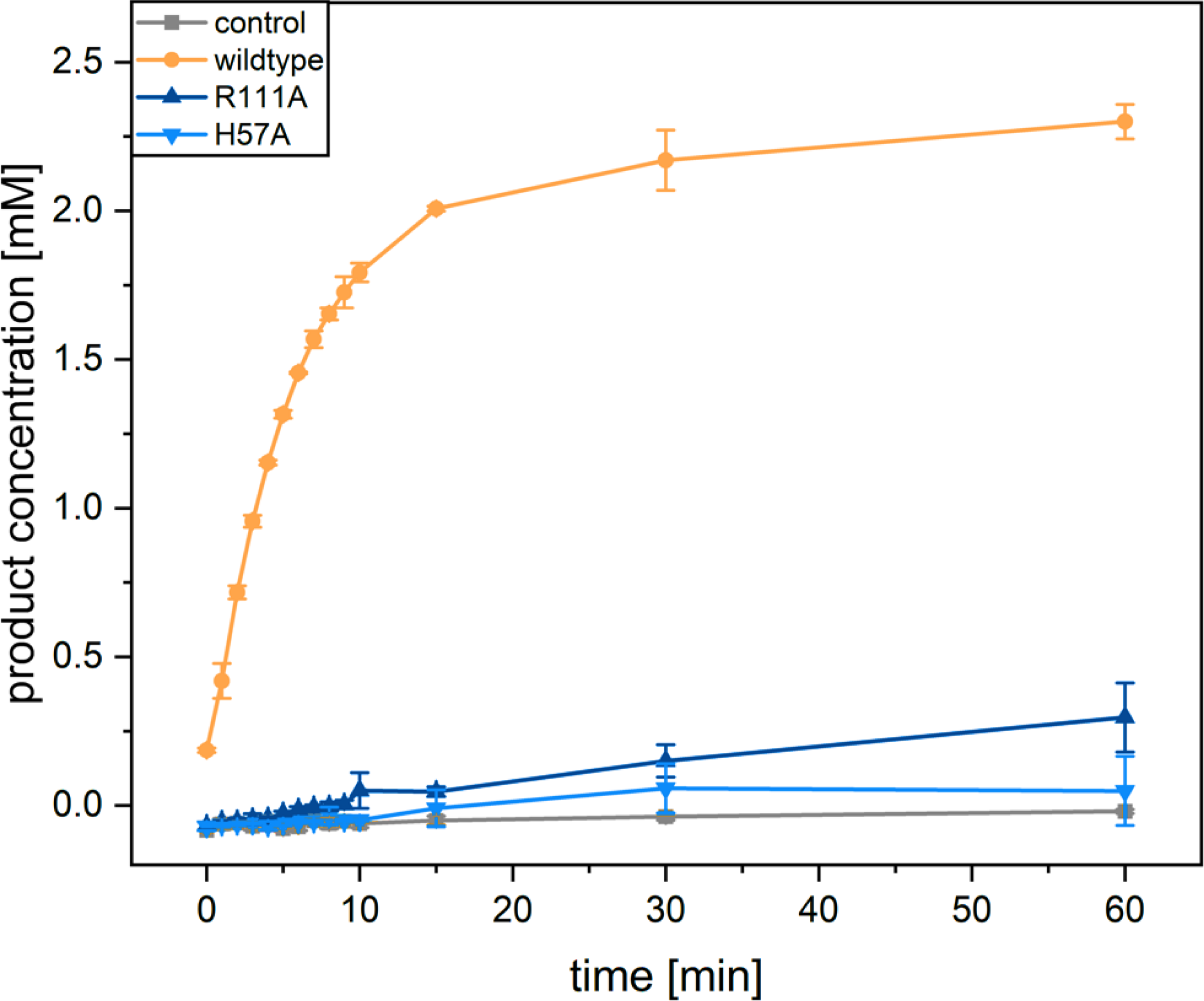
Activity assay of enriched membrane fraction. The curve indicates the amount of product 2-indanone produced over 60 min, measured at regular time intervals in RP-HPLC. The WT showed isomerization activity, whereas H57A showed no activity, and R111A showed several-fold reduced activity. The assay was performed in triplicate, and the data points indicate the average of the triplicate measurements, and the error bar indicates the standard deviation. The negative control was treated the same without a membrane fraction.

Enriched membrane fractions of *Ro*SOI1 variants showed similar activity profiles of whole-cell assays and retained their reddish color, except H57A (Fig. 5; Fig. S4 and S5). Interestingly, the R111A variant retained the red color, indicating the heme incorporation but showed significantly reduced activity, especially membrane purified, suggesting a structural role along with an unknown function in catalysis. UV/VIS spectra of R111A revealed a lower protein-to-heme ratio than the wild-type, supporting the hypothesis that this residue R111 interacts with the propionate group to stabilize the heme (Fig. S5). This hypothesis is consistent with the reported cryo-EM structure of SOI (17). The discrepancy in activity of the R111A variant in whole-cell assay to enriched or purified protein might be attributed to poorly coordinated heme in purified solution compared with the whole-cell system.

**Fig 6:**
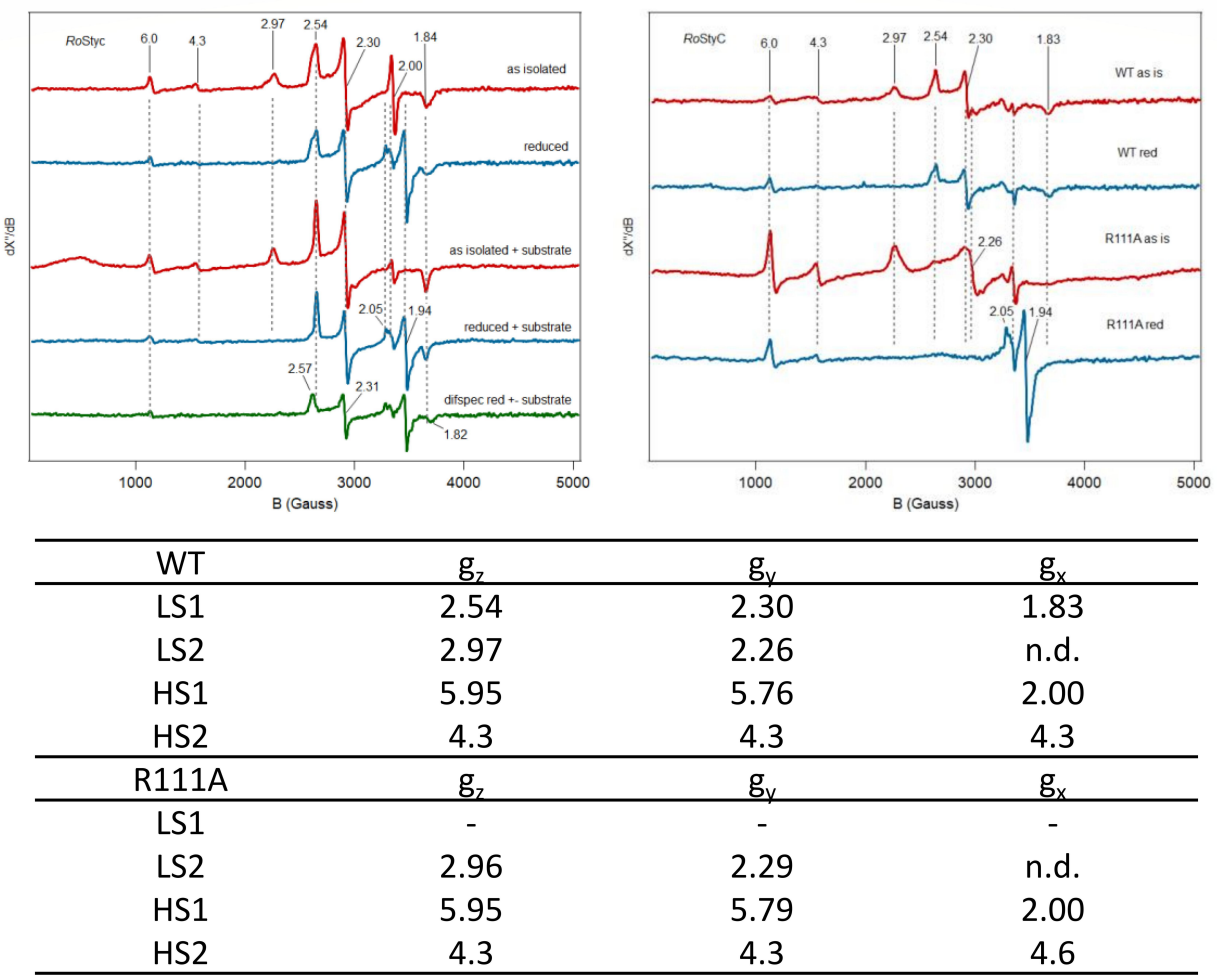
EPR spectroscopy. Both WT and R111A show his-his coordination. WT shows two low spin signals, only one of which reduced after titration with sodium dithionite, whereas R111A shows only one low spin that is reduced. The signals have been normalized to the maximal difference of the g_z_ and g_y_ signals of LS1. The spin signals and their corresponding *g*-values are given in the table. n.d – not detectable.

EPR spectroscopy further confirmed the differences in the heme environment. The WT exhibited two low-spin signals (LS1 and LS2) and one high-spin signal (HS). Only the LS2 signal (*g_z_* = 2.97) was reduced after the addition of 10 mM sodium dithionite, whereas LS1 was unaffected. The effect on HS is not consistent. LS2 is a broad signal with *g*-values indicative of nitrogenous ligands (bis-His). LS1 represents a heme with a different coordination environment, not both N-ligands. The fact that LS1 is not reduced suggests either that the redox potential is very low or that it is not accessible to exogenous ligands. Comparing *Ro*SOI1 WT and the R111A mutant, it is very clear that the Arg mutant only has LS2 and not LS1. Furthermore, the HS signal seems more prominent. The LS2 is also fully reduced after the addition of dithionite. The reduced R111A mutant shows an FeS cluster signal, which we attribute to a contaminating membrane protein. The increased HS and the *g* = 4.3 signal may arise from free heme and non-specifically bound Fe^3+^, respectively, due to precipitation observed in the R111A sample. The addition of substrate to both the oxidized and reduced WT samples results in the apparent sharpening of the LS1 and LS2 signals. Perhaps this could indicate the existence of two discrete protein conformations for heme in the purified enzyme and only one after turnover, or it could indicate substrate/product binding in the vicinity (but not directly coordinating) of the heme (Fig. 6).

This study demonstrates that *Ro*SOI1 can be functionally expressed as fusion proteins with SUMO, sfGFP, and mCherry. Biochemical and spectroscopic analyses, particularly H57A/C and R111A variants, confirm the essential role of H57 in axial heme coordination, establishing heme as a catalytic cofactor. EPR spectroscopy reveals the presence of different heme environments, whereas R111 appears to stabilize heme binding *via* interaction with the propionate group of the heme. These insights advance our understanding of SOI’s unique heme-based isomerization mechanism.

### Fluorescence microscopy of sfGFP-tagged SOI revealed its membrane localization

**Fig 7:**
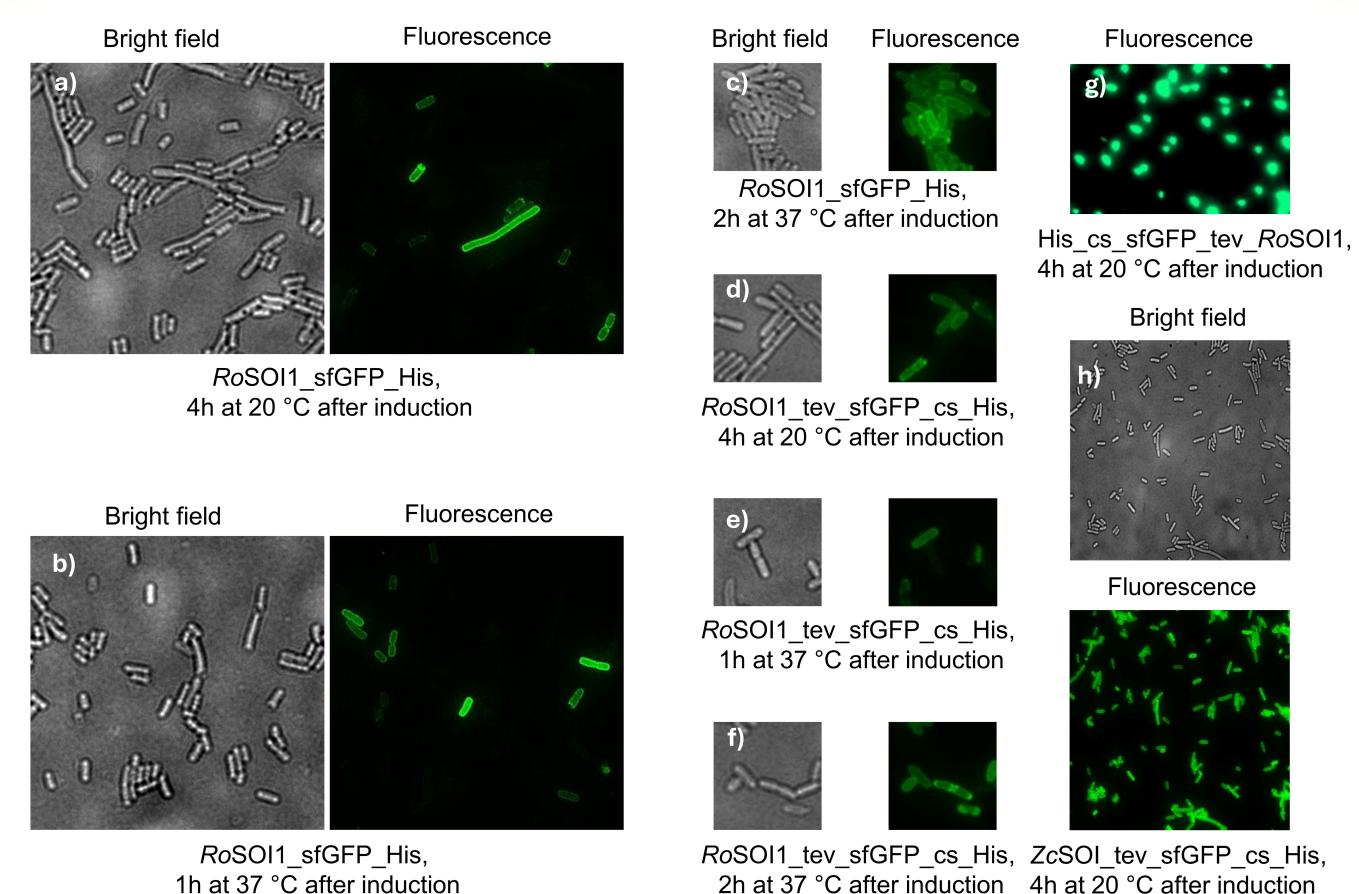
Fluorescence microscopy of sfGFP-tagged SOIs: Presented the bright field (left) and fluorescence (right) images. The images were taken with a 100× objective lens. For the GFP filter, an exposure time of 100 ms was used, and for the Brightfield filter, a time of 80 ms was used. (**a–c**) sfGFP fused at the *C-*terminal of *Ro*SOI1 without linkers, overproduction for 4 h at 20°C, 1 h at 37°C, 2 h at 37°C, respectively; (**d–f**) sfGFP fused at the *C-*terminal of *Ro*SOI1 with linkers, overproduction for 4 h at 20°C, 1 h at 37°C, 2 h at 37°C, respectively; (**g**) His_cs_sfGFP-tev-*Ro*SOI1, where sfGFP fused to the *N*-terminal. The overproduction of free sfGFP was visible in the cytosol. (**h**) The bottom image displays the fluorescence of sfGFP fused at the *C*-terminal of *Zc*SOI showing fluorescence around the membrane, whereas the top shows the brightfield image.

To determine the subcellular localization of SOI, cells expressing sfGFP- and mCherry-fused SOI were studied using fluorescence microscopy. The overproduction of *Ro*SOI1 with mCherry did not result in detectable fluorescence under a microscope, likely due to misfolding (data not shown). In contrast, sfGFP fused at the *N*-terminus of *Ro*SOI1 showed strong signals throughout the cells, which could be attributed to the accumulation of partially expressed fusion containing sfGFP protein (Fig. 7g). To overcome this, we examined functionally active, *C-*terminally tagged sfGFP fusions, expressed and observed under the microscope at different time points during overproduction. This exhibited distinct fluorescence localized at the cell membrane (Fig. 7a through f), suggesting proper expression and localization of the full-length fusion protein. Similar localization patterns were observed in *C-*terminally tagged sfGFP to *Zc*SOI from *Zavarzinia compransoris* Z-1155 (Fig. 7h). These microscopy studies revealed two important findings: SOIs are membrane-bound enzymes, and both the *N-* and *C-*termini are exposed to the cytosol, which is also evident from the available SOI structure (17).

To evaluate if the position of sfGFP influences expression and activity, we conducted overproduction, purification, and activity assays for various constructs. In the absence of any fusion tag, *Ro*SOI1 overexpression led to reddish-brown colored *E. coli* cell pellets, indicating the presence of heme. In contrast, sfGFP-tagged SOI constructs resulted in yellowish pellets, consistent with GFP expression.

**Fig 8:**
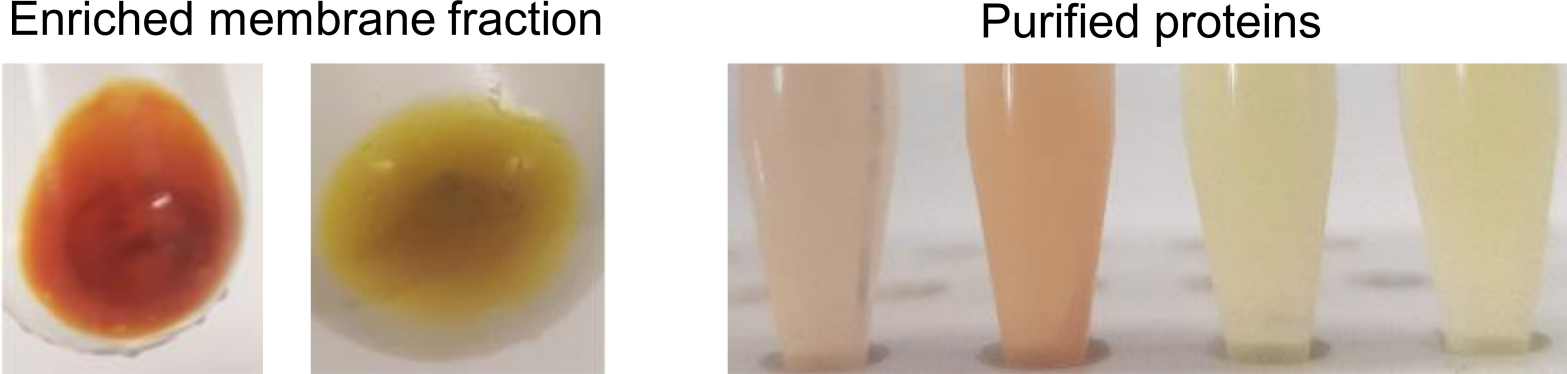
Enriched membrane fraction and purified *Ro*SOI1 proteins. Starting from left: non-solubilized fraction of His_cs_*Ro*SOI1, His_cs_sfGFP_tev_*Ro*SOI1. HisTrap purified *Ro*SOI1_cs_His, *Ro*SOI1_His, *Ro*SOI1_Tev_sfGFP_cs_His, and *Ro*SOI1_sfGFP_His. The protein concentrations are ~1 mg/mL, measured with the Bradford assay.

Purification by immobilized metal affinity chromatography yielded reddish-brown to greenish-brown proteins depending on the fusion (Fig. 8). Among all the constructs, the *N-*terminally tagged sfGFP fusion yielded the highest protein; however, since protein concentration was determined by Bradford assay, contributions from partially expressed His-tagged GFP cannot be completely excluded. In general, the heterologously produced SOI in *E. coli* yield ranged between 3 and 5 mg/L, with *Ro*SOI1_His producing only ~1 mg/L (Table S1).

Heme staining of SDS-PAGE gels showed bands corresponding to both monomeric and dimeric forms of the protein. Multiple bands corresponding to dimeric sizes were observed for SOIs earlier (16), and the oligomeric state of SOI was determined to be a trimer from our structural study (17). Interestingly, in the case of sfGFP fusions, heme staining revealed only the monomeric form, suggesting possible structural constraints imposed by the tag (Fig. S6).

UV/VIS spectra confirmed the presence of a heme Soret band at ~412 nm and at ~490 nm, which corresponds to the absorption maxima of GFP (Fig. S7). The activity assay using indene oxide as substrate showed that *Ro*SOI1 fused to either a His-tag or sfGFP at the *N-*terminal exhibited a maximum reaction rate of *k_obs_* ~ 8 s^−1^ for 1 mM indene oxide (Table 1; Fig. S10).

**TABLE 1:** The rate constant *k* and specific activity of differently tagged *Ro*SOI1 for 1 mM indene oxide as substrate are given^*a*^

Enzyme	Specific activity (μmoles mg^−1^min^−1^)	*k*_obs_ (s^−1^)
His_cs_*Ro*SOI1	25.3 ± 0.6	8.4 ± 0.2
His_cs_sfGFP_tev_*Ro*SOI1	10.2 ± 0.1	8.2 ± 0.1
*Ro*SOI1_cs_His	1.4 ± 0.03	0.5 ± 0.01
*Ro*SOI1_tev_sfGFP_cs_His	1.9 ± 0.03	1.5 ± 0.02
*Ro*SOI1_His	1.1 ± 0.05	0.4 ± 0.01
*Ro*SOI1_sfGFP_His	7.1 ± 0.2	5.4 ± 0.14

^*a*^ The assay was conducted at 25°C, 1,000 rpm. The values were calculated based on the amount of product (2-indanone) produced over time and quantified using RP-HPLC with an isocratic mobile phase of 60% acetonitrile and 40% dH_2_O with 0.1% TFA flowing through the C18-column at a flow rate of 0.7 mL min^−1^.

In contrast, the activity drastically reduced ~10-fold when tags were fused at the *C-*terminal, except GFP fusion without linkers (cs; cleavage site specific to Factor Xa and tev; TEV protease, were used as linkers in this study), retaining partial activity compared with WT. Even a short peptide like His_6_ with or without a cleavage site (cs) negatively impacted enzyme activity. This finding highlights the possible importance of the *C*-terminus for its contribution to proper substrate channeling. The sfGFP fusion without linkers showed relatively higher activity, which could be explained by the rigidity in fusion, maintaining or opening the substrate tunnel, compared with flexible linkers blocking it.

This phase of the study provides insights into the structure-function relations of *Ro*SOI1 and its fusion constructs. The use of the fluorescence microscopy technique with sfGFP demonstrated the membrane localization of SOI, with *N*- and *C*-termini localized in the cytosol. Functional assays revealed that the position and nature of tags significantly influence the activity of the enzyme, whereas the *N*-terminally fused tag did not alter or impair the activity, but the *C*-terminal tags negatively impacted catalysis, reinforcing the critical role of this region in maintaining the proper substrate tunnel architecture. Therefore, this made us focus on the *N-* and *C-*termini of SOIs.

### Truncation of the terminal extension in SOIs hints at its role in substrate channeling

A detailed multiple sequence alignment of SOIs displayed a conserved terminal extension pattern (Fig. S8), with most SOIs possessing either *N-*terminal or *C*-terminal extensions. Specifically, *Ro*SOI1 exhibits an 11-amino acid extension at its *C-*terminus, whereas *Zc*SOI harbors a 26-residue extension at the *N-*terminus. To investigate the functional significance of these terminal extensions, a series of truncated variants was constructed: N1-14_*Zc*SOI, N1-21_*Zc*SOI, and N1-32_*Zc*SOI for *Zc*SOI; and C164–167_*Ro*SOI1, C161–167_*Ro*SOI1, and C157–167_*Ro*SOI1 for *Ro*SOI1.

Western blot analysis of the enriched membrane fractions confirmed successful expression and membrane localization of all variants, ruling out the possibility that these extensions act as classical transit signal peptides. Furthermore, the reddish-brown color of the membrane fractions, along with heme staining, indicated that the heme cofactor was retained across all constructs (Fig. S9).

**Fig 9:**
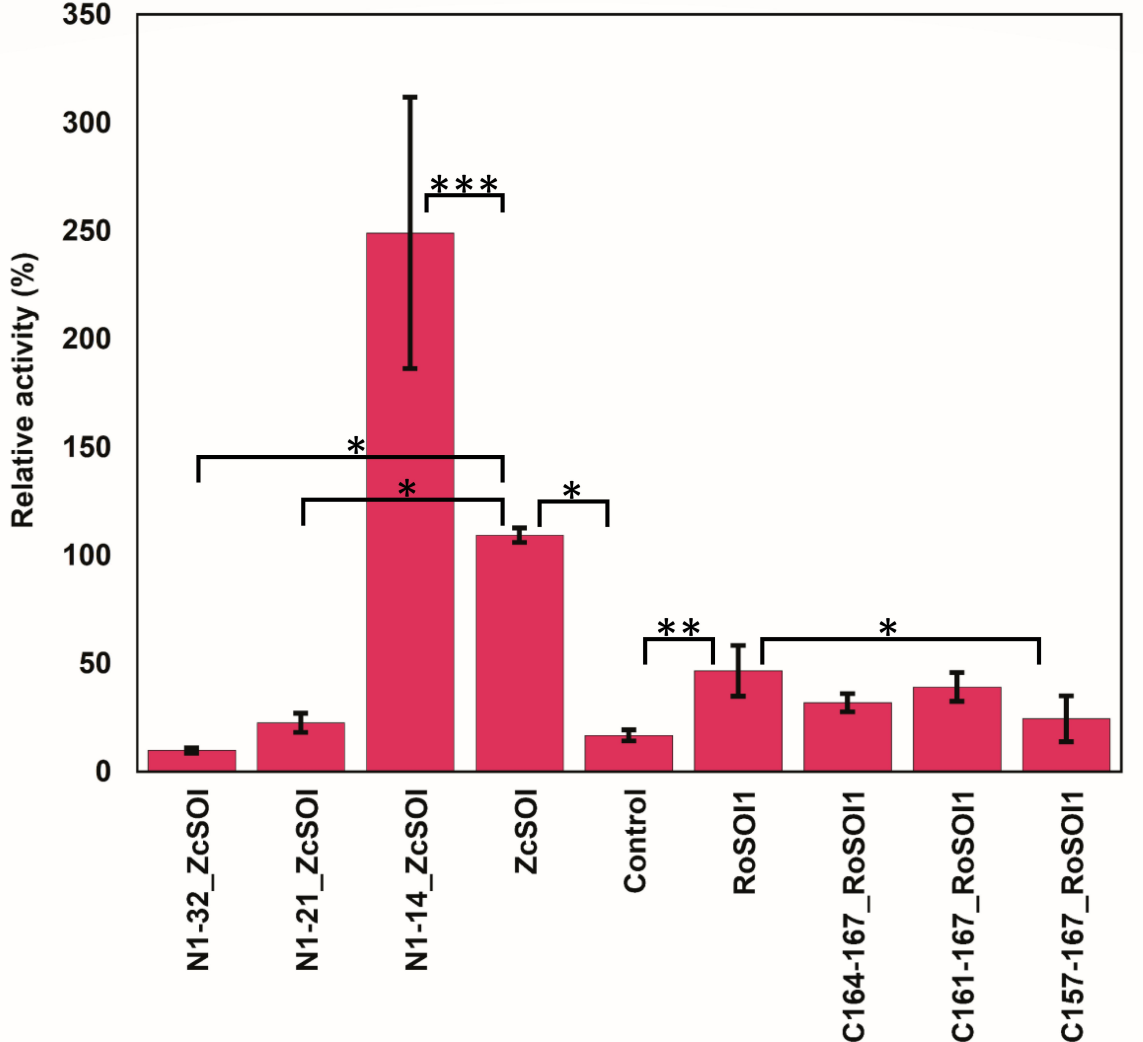
Activity assay of truncated SOIs for isomerization of styrene oxide. The bar indicates the average of three triplicates with error bars showing the standard deviation. The relative activity was calculated by accounting for *Zc*SOI’s activity as 100%. The one-way ANOVA presented significant differences with *P* < 0.0001 values. Furthermore, the post hoc test with Tukey HSD showed significant differences between *Zc*SOI and its truncated variants, especially N1-14_*Zc*SOI, which displayed ~2.5-fold increased activity compared with WT *Zc*SOI. On the contrary, N-1-21 and N1-32_*Zc*SOI variants showed severe loss in activity. On the other hand, less significance in difference was observed for *Ro*SOI1 and its corresponding truncated variants, indicating no drastic decrease in the activity. *P* < 0.05 given as (*), *P* < 0.001 given as (**), and *P* < 0.001 given as (***).

Activity assays using the natural substrate styrene oxide and quantification of the product phenylacetaldehyde revealed distinct roles of the terminal regions. All truncated *Ro*SOI1 variants displayed reduced activity relative to the wild-type. Interestingly, *Zc*SOI truncations N1-21 and N1-32 resulted in a complete loss of catalytic activity, whereas N1-14_*Zc*SOI exhibited a notable ~2.5-fold increase in activity compared with *Zc*SOI_WT (Fig. 9). These findings underscore the functional importance of the terminal extensions in catalysis, in addition to the preservation of membrane association and heme incorporation. It is likely that these regions contribute to efficient substrate channeling and potentially act as gatekeepers to the active site.

Moreover, given the proposed role of SOI as a membrane-anchored enzyme participating in a multi-step styrene degradation pathway, these terminal extensions may also mediate crucial protein–protein interactions with other enzymes in this pathway. Overall, the data might suggest the dual role of terminal overhangs in both catalytic efficiency and potential interaction with other proteins.

## DISCUSSION AND CONCLUSION

This study provides a functional and mechanistic characterization of styrene oxide isomerase *Ro*SOI1 from *Rhodococcus opacus* 1CP and *Zc*SOI from *Zavarzinia compransoris* Z-1155, involving fusion-based expression, site-directed mutagenesis to investigate the catalytic mechanism, membrane localization, and the role of *N-* and *C-*terminal extensions. Our holistic approach combining mutagenesis, biochemical assays, fluorescence microscopy, and spectroscopic analysis has provided new understanding into SOI’s structure-function relationship, particularly its unique heme-mediated catalysis.

The potential of SOI to be expressed as active fusion proteins with sfGFP and mCherry, along with previously reported SUMO-tag (16), enabled the functional characterization and its subcellular localization. The site-directed mutagenesis aimed at investigating the earlier hypothesized acid-base catalysis (14). Our findings, including the inactivity and loss of heme with H57A/C variants, firmly established that histidine-57 acts as the axial ligand for heme, possibly indicating the heme-mediated Lewis-acid catalysis, which was also observed and reported for SOI from *Pseudomonas* sp. VLB120 (17). The inability of cysteine in the variant H57C to rescue activity and heme loading underlines the importance of histidine coordination. R111A exhibited lower activity, but retained heme, and its instability suggests a structural role of R111, likely participating in interacting with the propionate moiety of the heme cofactor and not directly in catalysis. EPR spectroscopy further revealed at least two different environments for the heme cofactor with different low spin species (LS1 and LS2), of which one could not be chemically reduced, and the absence of LS1 in R111A variants reinforces its role in stabilizing the heme.

Fluorescence microscopy of the sfGFP-SOI fusions revealed that SOIs are a membrane-associated enzyme with both termini localized in the cytosol, proving the previously proposed hypothesis of SOI’s membrane localization (19). From the AlphaFold model, it is clear that the *N-/C-* terminal extensions are in close proximity to the proposed substrate tunnel (Fig. 1). Hence, the functional relevance of fusion tag positioning was further demonstrated through activity assays. *N*-terminally tagged constructs retained catalytic activity comparable with the wild-type enzyme, whereas *C*-terminal fusions, especially those incorporating flexible linkers, resulted in significantly reduced activity. Interestingly, rigid sfGFP fusions without linkers partially retained activity, possibly by stabilizing the substrate tunnel architecture. These findings highlight the functional sensitivity of the *C*-terminal region**,** which likely plays a role in substrate channeling, potentially by gating the active site. The role of terminal extensions in modulating substrate tunnel and activity is common in enzymes. For instance, the *N*-terminal extension of carbohydrate esterase is reported to control the substrate access into the active site by modulating the entrance tunnel (20).

Based on this, we explored the evolutionary conservation and functional role of terminal extensions in SOIs. Truncation of the 11-residue *C*-terminal extension in *Ro*SOI1 led to a reduction in activity, despite maintaining heme loading and membrane localization. More strikingly, truncation of the *N*-terminal extension in *Zc*SOI abolished activity entirely in longer truncations (N1–21 and N1–32), whereas a shorter truncation (N1–14) significantly enhanced activity. These results collectively indicate that the terminal overhangs serve critical functions, possibly in substrate gating or stabilization of active conformations. Moreover, their conserved nature and membrane localization of truncated variants suggest a potential role in protein–protein interactions**,** supporting our hypothesis that SOI operates as an anchor in an enzymatic complex in the styrene degradation pathway. As the heterologously produced SOIs in *E. coli* differ from the physiological environment of its natural host, the differences in activity of truncated variants in solution have to be dealt with care, as they might not depict the actual physiological conformation. However, our previous study on partial purification of SOI by enriching the membrane fraction from *Rhodococcus opacus* 1CP and *Sphingopyxis* sp. Kp5.2 (11, 19), and the similarities in activity data between partially purified (19) and solubilized enzyme (21) might support the closeness in enzyme topology. Despite this, more stringent care must be taken in the future to study and verify the protein-protein interaction of SOI with enzymes from the styrene degradation pathway.

## MATERIALS AND METHODS

### Multiple sequence alignment

Multiple sequence alignment of SOIs’ amino acid sequences was performed using Clustal Omega, and the consensus residues were visualized using WebLogo 3 (22, 23).

### Cloning, plasmid, and protein production

The primers used in this study are listed in Table S2. The fusion constructs with sfGFP, mCherry, and SUMO proteins were cloned into the pET-28a(+) vector using Gibson assembly. The genes and plasmids were amplified using PCR with 16 U/mL Q5 Hifi DNA polymerase (2000U/mL; New England BioLabs), ~100 ng plasmid or plasmid containing gene, 1.25 µL 10 pmol/µL of each primer, and 2 nM dNTP. The PCR was performed with 95°C initial denaturation for 2 min, 30 cycles of 95°C denaturation for 20 s, annealing for 20 s at 71°C, elongation for 3 min at 72°C, and final elongation for 10 min at 72°C. The successful amplification of PCR products was verified using 1% agarose gel electrophoresis. In addition, 8 µL of PCR products was digested with 1 µL of 10 U/µL DpnI in the presence of 1 µL 10 × cutsmart buffer overnight at 37°C. Later, it was subjected to Gibson assembly, for which 2 µL of gene fragment of interest, 1 µL of backbone, 3.75 µL Gibson master mix (NEBuilder HiFi DNA assembly master mix 2×) with 0.75 µL dH_2_O (deionized water) was incubated at 50°C for 1 h.

For site-directed mutagenesis, overlapping primers were used. The PCR was performed with 12.5 µL 2× PrimeStar Max premix (TaKaRa), 1 µL plasmid containing the gene of interest, 1.25 µL 10 pmol/µL of each primer, and 9 µL dH_2_O. PCR was performed with end-to-end primers to produce truncated SOIs, fusion constructs without linkers, and fusion constructs. The PCR conditions were the same as above, with denaturation for 15 sec and annealing for 15 sec (Table 2). Constructs produced with end-to-end primers were treated with 1 µL 10 × KLD enzyme mix, 5 µL 2 × KLD reaction buffer, 3 µL dH_2_O with 1 µL PCR product, and incubated at room temperature for 5 min. All the clones were verified using sequencing. The produced plasmids were transformed into *E. coli* DH5α and BL21/NiCo21 (DE3) for plasmid propagation and protein production, respectively. The proteins were overproduced, membrane fraction enrichment, and/or purification was performed as described in Kumaran et al. (21).

**TABLE 2:** PCR conditions for site-directed mutagenesis

Step		Time	Temperature
Initial denaturation		2 min	95^*a*^/ 98^*b*^^,^^*c*^°C
Denaturation	20^*a*^/ 25^*b*^/ 30^*c*^ cycles	15 sec	95^*a*^/ 98^*b*^^,^^*c*^°C
Annealing		15^*a*^/ 30^*b*^^,^^*c*^ sec	62^*a*^^,*b*^/ 50^*c*^°C
Elongation		3 min	72°C
Final elongation		10^*a*^/ 5^*b*^^,^^*c*^ min	72°C
Cooling		∞	10°C

^*a*^ Labels the PCR program used for producing the arginine variant.

^*b*^ Labels the PCR program used for producing aspartic acid, glutamic acid, and histidine variant.

^*c*^ Labels the PCR program used for producing a lysine variant.

### Determining the concentration of enriched membrane fraction and or purified protein via Bradford assay

The Bradford solution was prepared with a 5.5 parts deionized dH_2_O ratio to two parts roti-quant (5×). A BSA standard was prepared to calculate the membrane fraction concentration. The Bradford assay was performed in a 96-well plate for concentrations ranging between 0 and 200 µg mL^−1^. Therefore, each well contained 200 µL of the Bradford solution and 50 µL of the sample, or dH_2_O, for the blank value. The plate was incubated for 5 min at RT before measuring. The protein concentration was measured with OD_595_. To determine the exact concentration of samples, a 1:10, 1:50, and 1:100 dilution of the membrane fractions was prepared. Triplicates were made of each sample and each dilution to average the absorbance. To calculate the concentration of the samples in mg mL^1^, the average was substituted into the straight-line equation of the BSA standard.

### SDS-PAGE, western blot, and heme staining

SDS-PAGE was performed in a 12% resolving gel and a 5% stacking gel. Each well was loaded with 50 µg of the enriched membrane fractions; the PageRuler plus Prestained Protein Ladder (Thermo Scientific) was used as a marker. Before the samples were applied to the gel, 20 µL 2× SDS buffer was added and boiled at 95°C for 5 min. Then, the SDS-PAGE ran for 2 h at 100 V and 70 mA.

To verify if the SOIs were expressed, western blot detection was performed. Proteins were transferred from the polyacrylamide gel to a nitrocellulose membrane by western blotting and detected using an anti-His antibody. After SDS-PAGE was done, the gel was placed in the transfer buffer for approximately 20 min. Moreover, two sponges, one nitrocellulose membrane, and two sheets of Whatman filter paper (thickness 180 µm, diameter 8 × 10 cm) were soaked in the transfer buffer for around 10 min. The blotting sandwich was then run for one hour at 500 mA. The nitrocellulose membrane was blocked with 10 mL 1× TBS-T buffer with 3% wt/vol BSA at 4°C. Subsequently, the blocking solution was discarded, and the nitrocellulose membrane was washed three times for 5 min with 1× Tris-buffered saline with Tween 20 buffer (1× TBS-T buffer). Then, the nitrocellulose membrane was incubated for 2 h under constant shaking in 10 mL 1× TBS T buffer with 2.5 µL Penta-His HRP conjugate AK antibody to mark the SOI. The membrane was washed thrice with TBS-T buffer for 10 min. For detecting, 1 mL dH_2_O and 300 µL of Immobilon Forte Western HRP Substrate (Merck KGaA) were added to the membrane. The signals were detected by chemiluminescence using the chemiluminescence camera (FluorChem SP, Alpha Innotech).

Heme staining was performed to determine the presence of heme. Initially, an SDS-PAGE gel was prepared. The samples were prepared with 5 µL 4× SDS loading buffer and 15 µL of the protein sample, boiled at 90°C for 20 min. Then, 40–70 µg of protein was loaded onto the gel to reach the maximum possible protein amount for each pocket. Later, the gel was transferred into 80 mL of sodium acetate, pH 5.0, for 20 min on an orbital shaker, packed in aluminum foil. Continuing, 40 mg of TMBD was dissolved in 40 mL of methanol in an aluminum foil-covered centrifugal tube. The solution was poured over the gel with the sodium acetate. After incubation for 15 min maximum, 400 µL of hydrogen peroxide (30%) was added to the gel, and the bands were detected.

### Activity assay for whole-cell biocatalysis, membrane fractions, and purified enzyme

The whole-cell biocatalysis assay was performed for differently tagged and site-directed mutated SOI variants. The *E. coli* NiCo21 (DE3) cell pellet was resuspended in 0.1 M potassium phosphate buffer with a pH of 7.0. The reaction was performed in a 100 mL flask with a 10 mL reaction mixture containing a final concentration of 25 mM potassium phosphate buffer, pH 7.0, 1–2 mM indene oxide, and cells resuspended at OD_600_ of 10, and incubated at 25°C and 400 rpm for 60 min. Triplicates were made for each reaction sample. The assay was performed with a negative control (without cells). The reaction was started by adding the substrate. 100 µL of reaction mixture was quenched with 100 µL of stopping solution (acetonitrile with 0.2% vol/vol TFA). Samples were collected at regular time intervals (0 and 30 min, and every 1 h up to 6 h), vortexed, and centrifuged for 10 min at 17,000 × *g*. Then, 100 µL of the supernatants were transferred into RP-HPLC vials. For membrane fraction or purified protein, the assay was performed in 2 mL reaction tubes. The reaction mixture was prepared as described above with 1 µM membrane fraction or purified enzyme, incubated at 25°C and 1,000 rpm for 60 min. Samples were collected every minute from 0 to 10, 30, and 60 min and treated as mentioned before. The indene oxide and 2-indanone concentrations were quantified through RP-HPLC analysis. The mobile phase was isocratic with 60% acetonitrile and 40% dH_2_O with 0.1% TFA flowing through the column (Knauer Eurospher 100-5 C18 column, 125  ×  4 mm) at a flow rate of 0.7 mL min^−1^ for 6 min. The substrate and product concentration were measured at a UV/VIS profile at 205 nm and quantified with a proper standard treated the same. The product 2-indanone was eluted at the retention time of 3.9 ± 0.02 min.

For reactions using the substrate styrene oxide, the assay was performed as described in Kumaran et al. (21), with 25 nM protein for *Ro*SOI1 and truncated variants. The assay was performed in 1.5 mL glass vials with 1 mL reaction mixture containing 25 mM potassium phosphate buffer, pH 7.0, 0.005% wt/vol DDM, and 1 mM (*R/S*)-styrene oxide. The reaction was started by adding the substrate at 25°C, 1,000 rpm. 170 µL of reaction mixture was collected after 30 s and quenched with 2 µL 95% H_2_SO_4_, neutralized with 33 µL 2M NaOH, and finally, 135 µL methanol (HPLC-grade) was added and vortexed. The sample was prepared as described above and analyzed using RP-HPLC with the same C18 column containing an isocratic mobile phase of 50% methanol and 50% water +0.1% trifluoroacetic acid at a flow rate of 1 mL min^−1^ for 6 min. The substrate and product were measured at a UV/VIS profile at 206 nm and quantified against similarly treated standards. The substrate (*R/S*)-styrene oxide and product phenylacetaldehyde were eluted at the retention times of 1.8 ± 0.02 min and 3.7 ± 0.02 min, respectively. The experiment was performed in triplicate.

### Fluorescent microscopy

To determine the localization, the sfGFP/mCherry-tagged SOI containing pET28-a(+) plasmids were overexpressed in 1L autoinduction media. After 4 h of incubation at 20°C, the OD_600_ of the cell culture was measured, and 1 mL of the sample was taken. The sample was centrifuged at 6,000 × g, 5 min, the cell pellet was washed with 100 mM potassium phosphate buffer, pH 7.0, and the pellet was resuspended in 200 µL of the same buffer; 5 µL were transferred onto a microscope slide containing a 1.5% agarose coating. The sample was covered with a cover slip, and images of the cells were taken under the fluorescent microscope. The images were taken with a 100× objective lens and a Brightfield filter at 80 ms exposure time and a green/red filter with 100 ms exposure time.

### EPR spectroscopy

The EPR spectra were recorded on a Bruker EMXplus spectrometer with a helium-flow cryostat at 18 K. The following EPR conditions were used; microwave frequency, 9.410 GHz; microwave power, 20 mW; modulation frequency, 100 kHz; modulation amplitude, 20 G; temperature, 18K (24, 25).

### Statistical analysis

All the data presented in this manuscript have been evaluated using Microsoft Excel. The data represent the average of triplicates, and the error bar indicates the standard deviation unless mentioned otherwise. The statistical analysis was performed in KaleidaGraph Version 4.5.4 (26) using a one-way ANOVA (27) method for group comparison, which showed a significant difference in *P* < 0.0001. Hence, a post hoc test was conducted for each group (*Ro*SOI1 and its variants, and *Zc*SOI and its variants with control) separately with Tukey HSD, and 0.05 alpha threshold (28).

## Data Availability

The amino acid sequences of the proteins studied in this paper are available in the NCBI database under the accession number AII82581.1 (*Ro*SOI1), and TDP40089.1 (*Zc*SOI). The gene sequences of fusion tags and plasmid used in this study is provided in the supplementary material.

## SUPPLEMENTAL MATERIAL

Supplemental material spectrum.01526-25-s0001.docx Fig. S1 to S10; Tables S1 and S2.

ASM does not own the copyrights to Supplemental Material that may be linked to, or accessed through, an article. The authors have granted ASM a non-exclusive, world-wide license to publish the Supplemental Material files. Please contact the corresponding author directly for reuse.
