# Supplementary material for: Functional characterization and membrane localization of the styrene oxide isomerase from Rhodococcus opacus 1CP and Zavarzinia compransoris Z-1155
Source: Microbiol Spectr. 2025 Sep 12;13(10):e01526-25. doi: 10.1128/spectrum.01526-25 (PMC12502698; doi:10.1128/spectrum.01526-25)
Supplement: Supplemental material — Fig. S1 to S10; Tables S1 and S2. [file spectrum.01526-25-s0001.docx]

**Supplementary material**

**Functional characterization and membrane localization of the styrene oxide isomerase from *Rhodococcus opacus* 1CP and *Zavarzinia compransoris* Z-1155**

Selvapravin Kumaran,^a^ Shanice Olanipekun,^a,*^ Latife Sönmez,^a,§^ Lars Janzen,^a^ Peter-Leon Hagedoorn,^b^ Dirk Tischler,^a,#^

^a^Microbial Biotechnology, Ruhr University Bochum, Bochum, Germany.

^b^Department of Biotechnology, Delft University of Technology, Delft, The Netherlands.

Running Head: Functional characterization of styrene oxide isomerase

^#^Address correspondence to Dirk Tischler, [dirk.tischler@email.de](mailto:dirk.tischler@email.de)

^*^Present address: Chair of Technical Biochemistry, Technical University Dortmund, Dortmund, Germany.

^§^Present address: Cellular Neurobiology, Department of Biology and Biotechnology, Ruhr University Bochum, Bochum, Germany.

Shanice Olanipekun and Latife Sönmez contributed equally to this work. Author order was decided by mutual consultation.

**Keywords:** Integral membrane protein, fluorescence microscopy, site-directed mutagenesis, EPR, sfGFP/SUMO, cytosolic termini, membrane anchor, terminal extension, truncation.


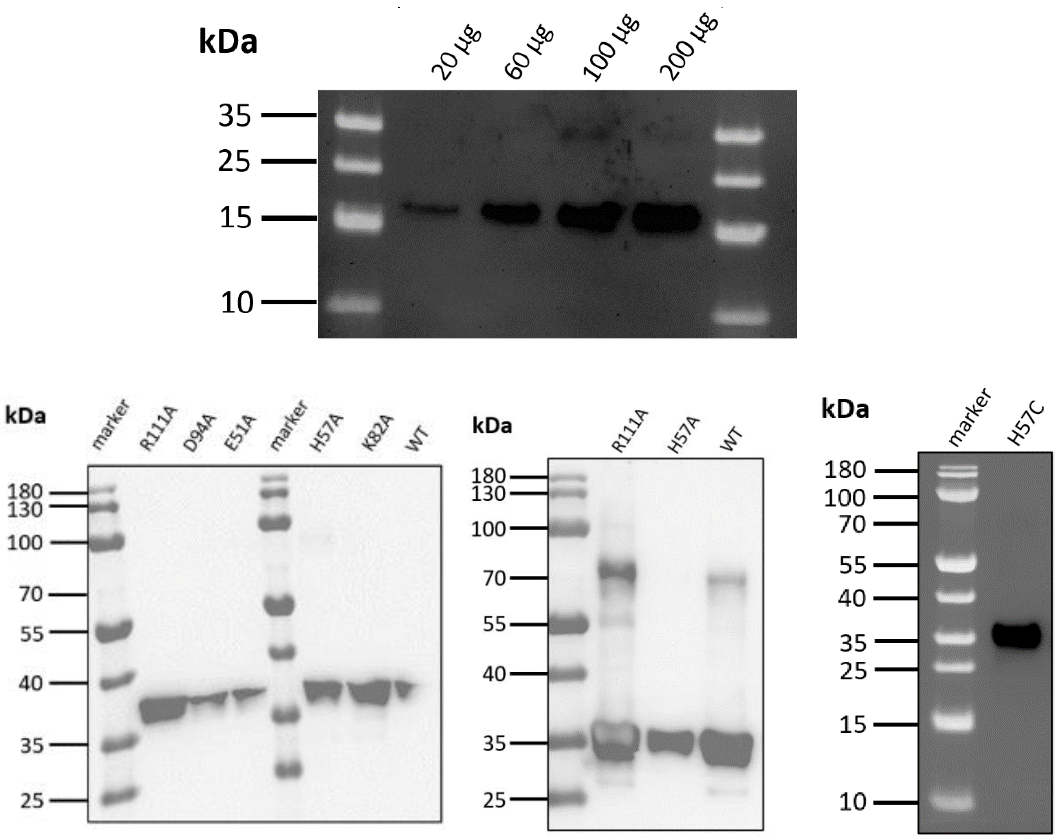


FIG S1: Western blot of *Ro*SOI1 fusions and variants. The bands that correspond to the monomeric and or dimeric size of *Ro*SOI1 WT without tag (top) and site-directed mutagenetic variants (bottom) fused to SUMO were visible between 35-40 kDa (Calculated size of SUMO-fused *Ro*SOI1; 32.2 kDa). As marker a PageRuler plus^TM^ Prestained Protein Ladder (Thermo Scientific) was used.


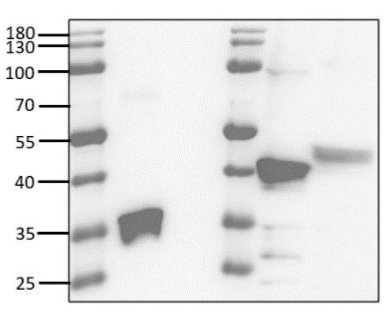


FIG S2: Western blot of *Ro*SOI1 fusions with different tags. Lane 1 - marker, lane 2 - SUMO fused *Ro*SOI1, lane 3 – marker, lane 4 – sfGFP fused *Ro*SOI1, lane 5 – mCherry fused *Ro*SOI1 (Calculated size of SUMO- *Ro*SOI1; 32.2 kDa), sfGFP-*Ro*SO; 49.97, mCherry-*Ro*SO; 49.92. As a marker, a PageRuler plus^TM^ Prestained Protein Ladder (Thermo Scientific) was used.


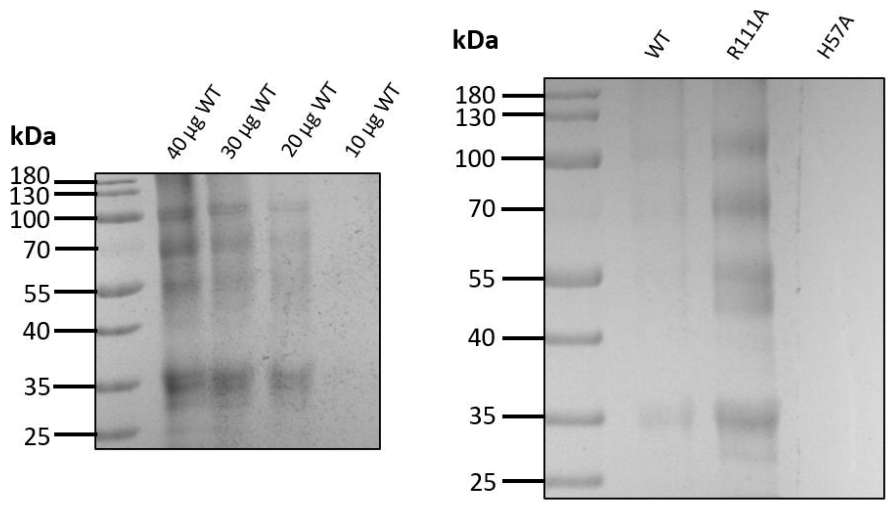


FIG S3: Heme staining of *Ro*SOI1 samples. Multiple bands that correspond to monomeric, dimeric, and trimeric sizes were observed. As marker a PageRuler plus^TM^ Prestained Protein Ladder (Thermo Scientific) was used.


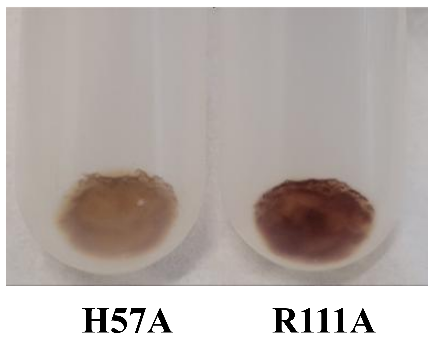


FIG S4: Enriched membrane fraction. The pellet of the membrane fractions of *Ro*SOI1 H57A (left) shows pale colour, and the R111A variant (right) with reddish-brown colour.


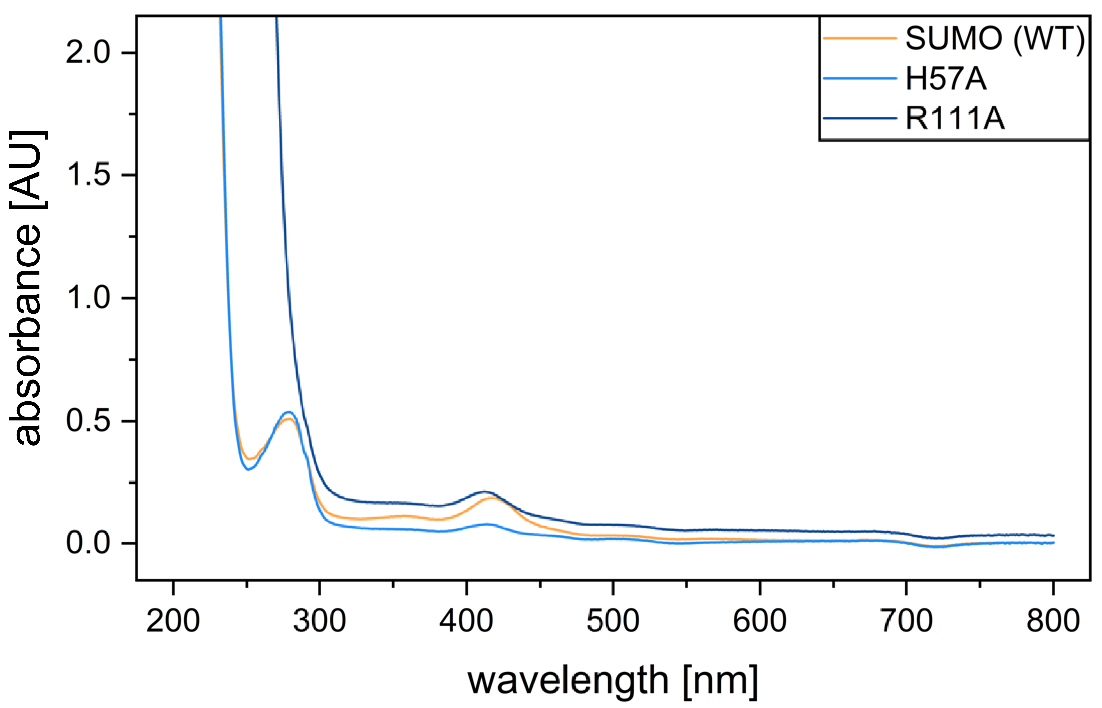


FIG S5: UV/VIS spectra of purified *Ro*SOI fusion and its variants. The WT and variants R111A showed a Soret band at around 412 nm while H57A showed no band in that range.


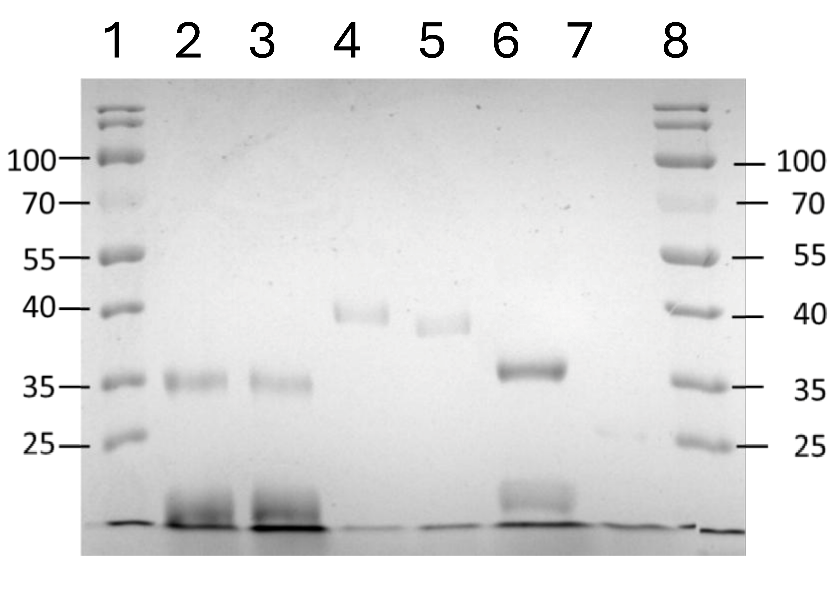


FIG S6: Heme staining of *Ro*SOI1 fused with sfGFP with and without linkers. Bands that correspond to the monomeric and dimeric size of respective proteins were visible for SOIs without sfGFP and only monomer for SOI with GFP. (lane 1 – marker, lane 2 – *Ro*SOI1_cs_His , lane 3 – *Ro*SOI1_His, lane 4 – *Ro*SOI1_tev_sfGFP_cs_His, lane 5 – *Ro*SOI1_sfGFP_His, lane 6 – His_cs_*Ro*SOI1, lane 7 – His_cs_sfGFP_tev_*Ro*SOI1, lane 8 – marker)


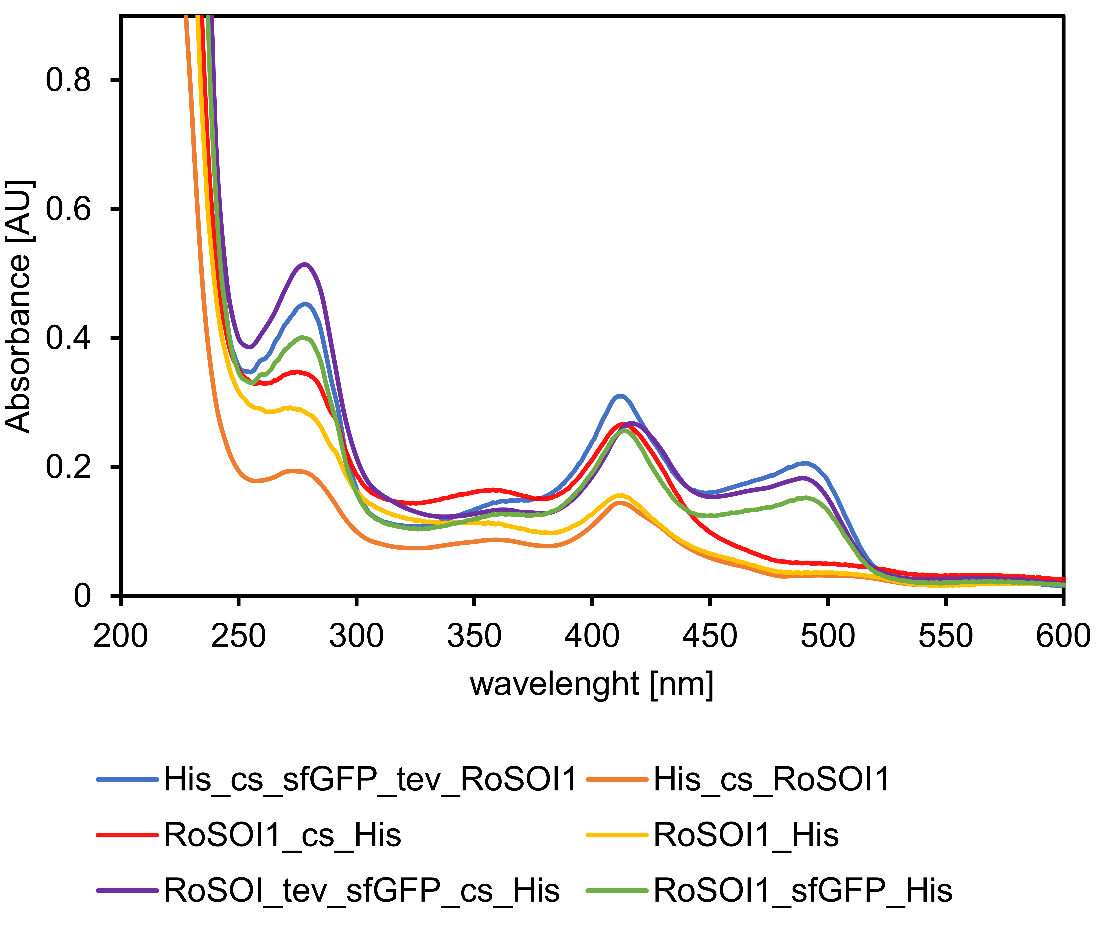


FIG S7: UV/VIS spectra of the differently tagged *Ro*SOI1 proteins. The absorbance spectrum of 10 µM purified protein was recorded between 200 nm and 800 nm. The heme has an absorption maximum of around 412 nm, and the sfGFP around 490 nm.


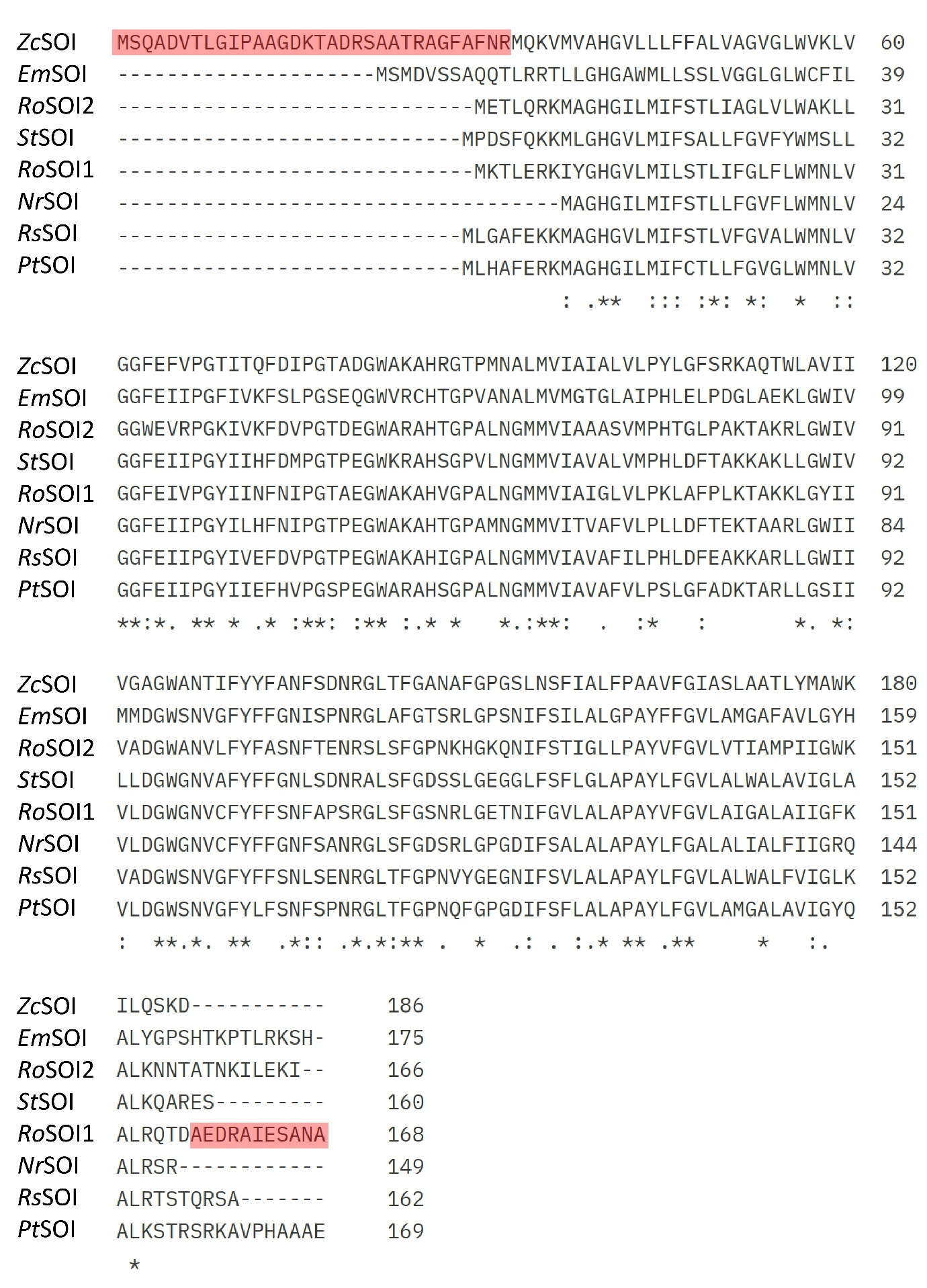
FIG S8 Multiple sequence alignment of SOIs showing terminal extensions. The extended amino acids are highlighted in red.


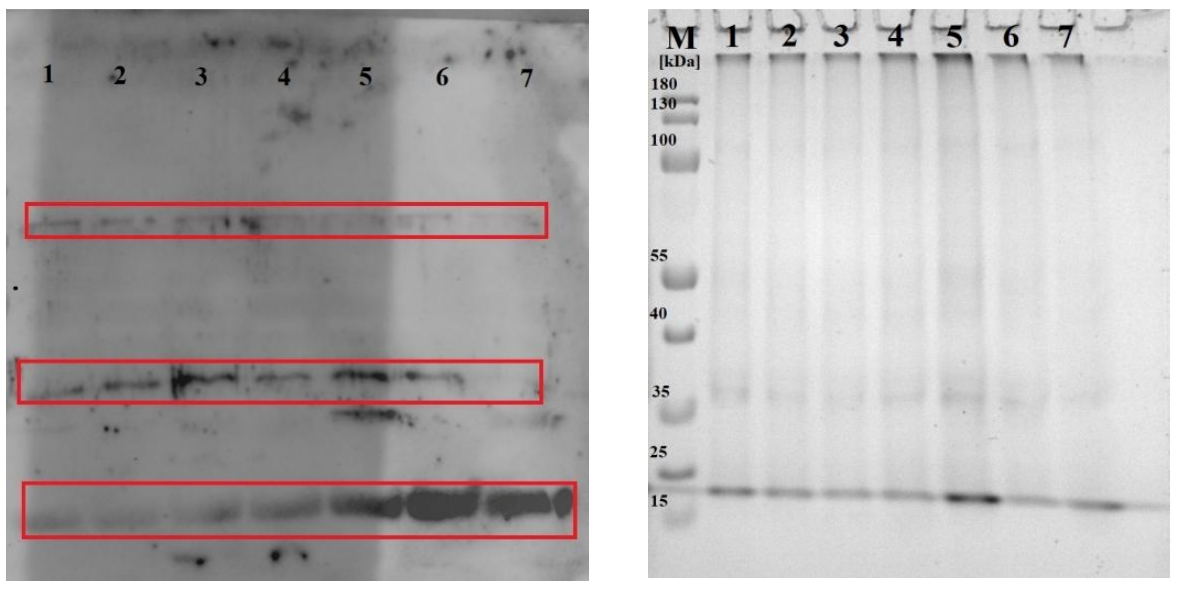


FIG S9 Western blot and heme staninig of truncated SOIs. The western blot showed the presence of three bands that corresponds to monomer, dimer and trimer while only monomeric sized bands were observed for heme staining. The lanes from left to right contain Protein Ladder (M), *Ro*SOI1 (1), C164-167*_Ro*SOI1 (2), C161-167*_Ro*SOI1 (3), C157-167*_Ro*SOI1 (4), *Zc*SOI (5), N1-32*_Zc*SOI (6), N1-14*_Zc*SOI (7).


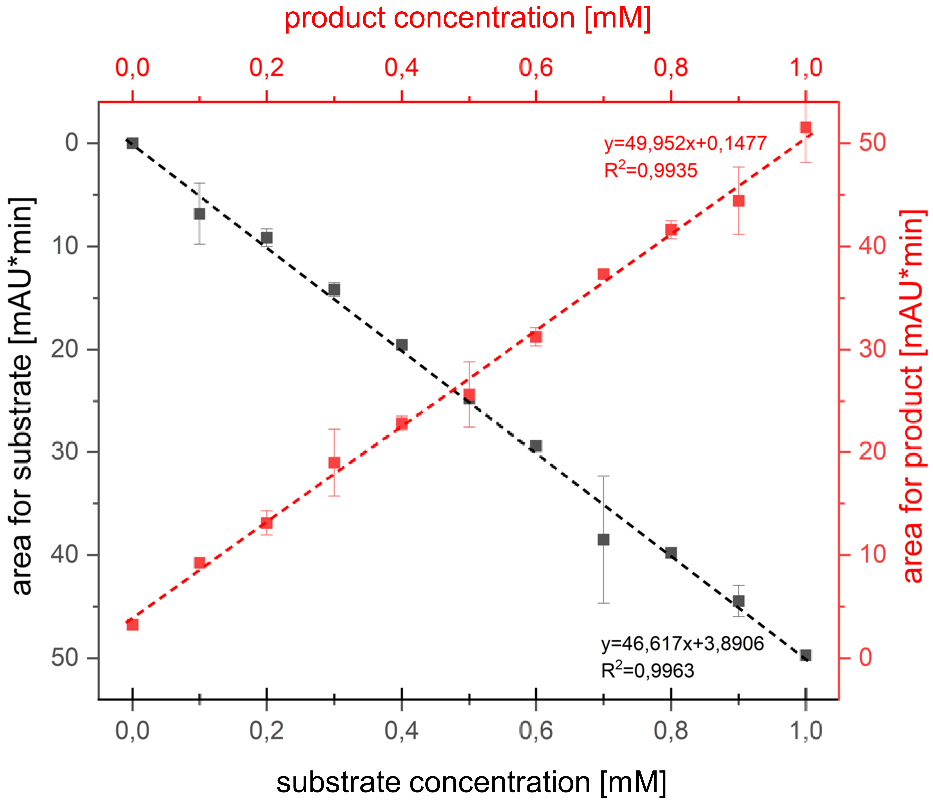


FIG S10 RP-HPLC calibration curve for substrate and product quantification (indene oxide and 2-indanone). Substrate and Product are given in black and red and the corresponding area is given in primary and secondary y-axis respectively. The X-axes shows the concentration and Y-axes shows the area observed in the RP-HPLC.

**TABLE S1 Total protein amount of the purification fractions.**

| **Protein** | **OD_600_** | **Cytosolic fraction [mg]** | **Tween 20 solubilized fraction [mg]** | **Flow-through [mg]** | **Wash 1/ wash 2 [mg]** | **Purified protein [mg]** |
| --- | --- | --- | --- | --- | --- | --- |
| *Ro*SOI1_cs_His | 4.73 | 511.83 | 88.55 | 70.62 | 15.55 | 2.85 |
| *Ro*SOI1_His | 3.01 | 275.47 | 60.39 | 38.24 | 5.74 | 0.82 |
| *Ro*SOI1_tev_sfGFP_cs_His | 4.18 | 364.80 | 45.44 | 55.44 | 7.46 | 2.94 |
| *Ro*SOI1_sfGFP_His | 2.64 | 473.53 | 68.70 | 50.51 | 28.73 | 10.17 |
| His_cs_ *Ro*SOI1 | *NA* | | | | | 5.2 |
| His_cs_sfGFP_tev_ *Ro*SOI1 |  |  |  |  |  | 32.1 |

**TABLE S2 List of primers used in this study**

| **Primers for site-directed mutagenesis in His_cs_SUMO_tev_*Ro*SOI1** | | | |
| --- | --- | --- | --- |
| Primers | | Sequence 5’ to 3’ | length |
| R111A | Fw | AACTTTGCTCCATCTGCCGGTCTATCTTTTGGC | 33 |
|  | Rev | GCCAAAAGATAGACCGGCAGATGGAGCAAAGTT | 33 |
| D94A | Fw | TACATTATCGTATTAGCCGGTTGGGGTAATGTC | 33 |
|  | Rev | GACATTACCCCAACCGGCTAATACGATAATGTA | 33 |
| E51A | Fw | ATTCCAGGTACAGCTGCAGGTTGGGCTAAAGCT | 33 |
|  | Rev | AGCTTTAGCCCAACCTGCAGCTGTACCTGGAAT | 33 |
| H57A | Fw | GGTTGGGCTAAAGCTGCCGTTGGTCCTGCTTTA | 33 |
|  | Rev | TAAAGCAGGACCAACGGCAGCTTTAGCCCAACC | 33 |
| K82A | Fw | TTAGCATTCCCGTTAGCAACTGCAAAAAAACTT | 33 |
|  | Rev | AAGTTTTTTTGCAGTTGCTAACGGGAATGCTAA | 33 |
| H57C | Fw | GGTTGGGCTAAAGCTTGCGTTGGTCCTGCTTTA | 33 |
|  | Rev | TAAAGCAGGACCAACGCAAGCTTTAGCCCAACC | 33 |
| T7 | Fw | TAATACGACTCACTATAGGG | 20 |
| T7term | Rev | TGCTAGTTATTGCTCAGCGG | 20 |
| **Primers for fusion construct** | | |  |
| 1. **Construct His_cs_*Ro*SOI1 in pET28-a(+) vector** | | | |
| To amplify pET28-a(+) vector for cloning *Ro*SOI1 | | | |
| pET28a Gib | Fw | AAGCTTGCGGCCGCACTCGA | 20 |
| pET28aGib | Rev | ATGGCTGCCGCGCGGCAC | 18 |
| To amplify *Ro*SOI1 with overhangs of cs and pET28-a(+) | | | |
| Rhod_28 Gib | Fw | AGCAGCGGCCTGGTGCCGCGCGGCAGCCATATGGGAATGAAAACACTGGA | 50 |
| Rhod_28 Gib | Rev | GTGGTGGTGCTCGAGTGCGGCCGCAAGCTTTCATGCATTAGCACTTTCAA | 50 |
| 1. **Construct His_cs_sfGFP_tev_*Ro*SOI1 in pET28-a(+) vector** | | | |
| To amplify pET28-a(+) vector containing *Ro*SOI1 using construct 1 as template | | | |
| 28a_Fw | Fw | AGCAGCGAGAACCTGTACTTCCAGGGACATATGGGAATGAAAACACTGGA | 50 |
| 28a_Rev | Rev | ATGGCTGCCGCGCGGCACCAG | 21 |
| To amplify *sf*GFP with overhangs of cs and tev | | | |
| His_GFP | Fw | AGCAGCGGCCTGGTGCCGCGCGGCAGCCATATGAGCAAAGGAGAAGAACT | 50 |
| GFP_Tev | Rev | ATGTCCCTGGAAGTACAGGTTCTCGCTGCTTTTGTAGAGCTCATCCATGC | 50 |
| 1. **Construct His_cs_mCherry_tev_*Ro*SOI1 in pET28-a(+) vector** | | | |
| To amplify pET28-a(+) vector containing *Ro*SOI1 using construct 1 as template | | | |
| 28a_Fw | Fw | AGCAGCGAGAACCTGTACTTCCAGGGACATATGGGAATGAAAACACTGGA | 50 |
| 28a_Rev | Rev | ATGGCTGCCGCGCGGCACCAG | 21 |
| To amplify mCherry with overhangs of cs and tev | | | |
| His_mCherry | Fw | AGCAGCGGCCTGGTGCCGCGCGGCAGCCATATGGTGAGCAAGGGCGAGGA | 50 |
| mCherry_Tev | Rev | ATGTCCCTGGAAGTACAGGTTCTCGCTGCTCTTGTACAGCTCGTCCATGC | 50 |
| 1. **Construct His_cs_SUMO_tev_*Ro*SOI1 in pET28-a(+) vector** | | | |
| To amplify pET28-a(+) vector containing *Ro*SOI1 using construct 1 as template | | | |
| 28a_Fw | Fw | AGCAGCGAGAACCTGTACTTCCAGGGACATATGGGAATGAAAACACTGGA | 50 |
| 28a_Rev | Rev | ATGGCTGCCGCGCGGCACCAG | 21 |
| To amplify SUMO with overhangs of cs and tev | | | |
| His_Sumo | Fw | AGCGGCCTGGTGCCGCGCGGCAGCCATGACTCAGAAGTCAATCAAGAAGC | 50 |
| Sumo_Tev | Rev | TGTCCCTGGAAGTACAGGTTCTCGCTGCTACCTCCAATCTGTTCGCGGTG | 50 |
| 1. **Construct *Ro*SOI1_cs_His in pET28-a(+) vector** | | | |
| To amplify pET28-a(+) vector with overhangs of cs and *Ro*SOI1 using construct 1 | | | |
| cs_pET28a | Fw | TGGTGCCGCGCGGCAGCCATCACCACCACCACCACCACTGAGATCCGGCT | 50 |
| *Ro*SOI1_pET28a | Rev | TCCAGTGTTTTCATTCCCATGCTGCTGCCCATGGTATATC | 40 |
| To amplify *Ro*SOI1 with overhangs of cs and pET28-a(+) | | | |
| pET28a_RoSOI1 | Fw | GATATACCATGGGCAGCAGCATGGGAATGAAAACACTGGA | 40 |
| cs_*Ro*SOI1 | Rev | CGCGGCACCAGGCCGCTGCTTGCATTAGCACTTTCAATAGC | 41 |
| 1. **Construct *Ro*SOI1_tev_sfGFP_cs_His in pET28-a(+) vector** | | | |
| To amplify pET28-a(+) vector containing *Ro*SOI1 with overhangs of tev and sfGFP using construct 5 as the template | | | |
| *Ro*SOI1_  Tev_GFP | Fw | AGCAGCGAGAACCTGTACTTCCAGGGACATATGAGCAAAGGAGAAGAAC | 49 |
| Tev_StyC | Rev | AAGTACAGGTTCTCGCTGCTTGCATTAGCACTTTCAATAGCACGATCTTC | 50 |
| To amplify sfGFP gene with overhangs of tev and cs | | | |
| *Ro*SOI1_GFP_cs_tev | Fw | ATGGATGAGCTCTACAAAAGCAGCGGCCTGGTGCCGCGCGGCAGCCAT | 48 |
| cs_GFP | Rev | CGCGGCACCAGGCCGCTGCTTTTGTAGAGCTCATCCATGC | 40 |
| 1. **Construct *Ro*SOI1_sfGFP_His in pET28-a(+) vector** | | | |
| To amplify pET28-a(+) vector containing *Ro*SOI1 without cs, with sfGFP overhang using construct 5 as the template | | | |
| GFP_His | Fw | GCATGGATGAGCTCTACAAACACCACCACCACCACCACTG | 40 |
| GFP_StyC | Rev | AGTTCTTCTCCTTTGCTCATTGCATTAGCACTTTCAATAGCACG | 44 |
| To amplify sfGFP with overhangs of His and *Ro*SOI1 | | | |
| *Ro*SOI1_GFP | Fw | CTATTGAAAGTGCTAATGCAATGAGCAAAGGAGAAGAACT | 40 |
| His_GFP | Rev | CAGTGGTGGTGGTGGTGGTGTTTGTAGAGCTCATCCATGC | 40 |
| 1. **Construct *Ro*SOI1_His in pET28-a(+) vector** | | | |
| To amplify pET28-a(+) vector containing *Ro*SOI1 without any overhangs using construct 7 | | | |
| pET28a_His | Fw | CACCACCACCACCACCACTGAGATCC | 26 |
| pET28a_StyC | Rev | TGCATTAGCACTTTCAATAGCACG | 24 |
| 1. **Construct *Zc*SOI_tev_sfGFP_cs_His in pET28-a(+) vector** | | | |
| To amplify pET28-a(+) containing sfGFP using construct 6 as template | | | |
| *Ro*SOI1_Tev_GFP | Fw | AGCAGCGAGAACCTGTACTTCCAGGGACATATGAGCAAAGGAGAAGAAC | 49 |
| *Ro*SOI1_GFP_cs_tev | Rev | ATGGATGAGCTCTACAAAAGCAGCGGCCTGGTGCCGCGCGGCAGCCAT | 48 |
| To amplify *Zc*SOI gene with overhangs of tev and cs | | | |
| *Zc*SOI_pET28a | Fw | GATATACCATGGGCAGCAGCATGGGAATGAGTCAAGCAGACGTTACATTG | 50 |
| *Zc*SOI _Tev | Rev | ATGTCCCTGGAAGTACAGGTTCTCGCTGCTCTCGAGGTCTTTTGATTG | 48 |
| **Truncated variants** | | | |
| pET28-a(+) containing *Zc*SOI with C-termini 6x His was used as template | | | |
| N1-21 | Fw | ATGGCTGCAACACGTGCGGGTTT | 23 |
| N1-14 | Fw | ATGGATAAAACAGCGGATCG | 20 |
| N1-32 | Fw | ATGCAGAAGGTCATGGTAG | 19 |
| N_trunc | Rev | GGCCATGGTATATCTCCTTC | 20 |
| Construct 1 was used as the template | | | |
| C164-167 | Rev | TTCAATAGCACGATCTTCAGCATC | 24 |
| C161-167 | Rev | ACGATCTTCAGCATCAGTTTGACG | 24 |
| C157-167 | Rev | ATCAGTTTGACGAAGAGCCTTAAAACC | 27 |
| C-trunc | Fw | TGAAAGCTTGCGGCCGCACTCGAG | 24 |

**Gene sequences:**

**sfGFP**

ATGAGCAAAGGAGAAGAACTTTTCACTGGAGTTGTCCCAATTCTTGTTGAATTAGATGGTGATGTTAATGGGCACAAATTTTCTGTCCGTGGAGAGGGTGAAGGTGATGCTACAAACGGAAAACTCACCCTTAAATTTATTTGCACTACTGGAAAACTACCTGTTCCGTGGCCAACACTTGTCACTACTCTGACCTATGGTGTTCAATGCTTTTCCCGTTATCCGGATCACATGAAACGGCATGACTTTTTCAAGAGTGCCATGCCCGAAGGTTATGTACAGGAACGCACTATATCTTTCAAAGATGACGGGACCTACAAGACGCGTGCTGAAGTCAAGTTTGAAGGTGATACCCTTGTTAATCGTATCGAGTTAAAGGGTATTGATTTTAAAGAAGATGGAAACATTCTTGGACACAAACTCGAGTACAACTTTAACTCACACAATGTATACATCACGGCAGACAAACAAAAGAATGGAATCAAAGCTAACTTCAAAATTCGCCACAACGTTGAAGATGGTTCCGTTCAACTAGCAGACCATTATCAACAAAATACTCCAATTGGCGATGGCCCTGTCCTTTTACCAGACAACCATTACCTGTCGACACAATCTGTCCTTTCGAAAGATCCCAACGAAAAGCGTGACCACATGGTCCTTCTTGAGTTTGTAACTGCTGCTGGGATTACACATGGCATGGATGAGCTCTACAAA

**Cleavage site (cs)**

AGCAGCGGCCTGGTGCCGCGCGGCAGCCAT

**Histidine tag (His)**

CACCACCACCACCACCAC

**Tev protease (tev)**

AGCAGCGAGAACCTGTACTTCCAGGGACAT

**SUMO** GACTCAGAAGTCAATCAAGAAGCTAAGCCAGAGGTCAAGCCAGAAGTCAAGCCTGAGACTCACATCAATTTAAAGGTGTCCGATGGATCTTCAGAGATCTTCTTCAAGATCAAAAAGACCACTCCTTTAAGAAGGCTGATGGAAGCGTTCGCTAAAAGACAGGGTAAGGAAATGGACTCCTTAAGATTCTTGTACGACGGTATTAGAATTCAAGCTGATCAGGCCCCTGAAGATTTGGACATGGAGGATAACGATATTATTGAGGCTCACCGCGAACAGATTGGAGGT

**mCherry** ATGGTGAGCAAGGGCGAGGAGGATAACATGGCCATCATCAAGGAGTTCATGCGCTTCAAGGTGCACATGGAGGGCTCCGTGAACGGCCACGAGTTCGAGATCGAGGGCGAGGGCGAGGGCCGCCCCTACGAGGGCACCCAGACCGCCAAGCTGAAGGTGACCAAGGGTGGCCCCCTGCCCTTCGCCTGGGACATCCTGTCCCCTCAGTTCATGTACGGCTCCAAGGCCTACGTGAAGCACCCCGCCGACATCCCCGACTACTTGAAGCTGTCCTTCCCCGAGGGCTTCAAGTGGGAGCGCGTGATGAACTTCGAGGACGGCGGCGTGGTGACCGTGACCCAGGACTCCTCCCTGCAGGACGGCGAGTTCATCTACAAGGTGAAGCTGCGCGGCACCAACTTCCCCTCCGACGGCCCCGTAATGCAGAAGAAGACCATGGGCTGGGAGGCCTCCTCCGAGCGGATGTACCCCGAGGACGGCGCCCTGAAGGGCGAGATCAAGCAGAGGCTGAAGCTGAAGGACGGCGGCCACTACGACGCTGAGGTCAAGACCACCTACAAGGCCAAGAAGCCCGTGCAGCTGCCCGGCGCCTACAACGTCAACATCAAGTTGGACATCACCTCCCACAACGAGGACTACACCATCGTGGAACAGTACGAACGCGCCGAGGGCCGCCACTCCACCGGCGGCATGGACGAGCTGTACAAG

**Plasmid sequence (pET28-a(+)_His_cs_SUMO_tev_*Ro*SOI1)**

TGGCGAATGGGACGCGCCCTGTAGCGGCGCATTAAGCGCGGCGGGTGTGGTGGTTACGCGCAGCGTGACCGCTACACTTGCCAGCGCCCTAGCGCCCGCTCCTTTCGCTTTCTTCCCTTCCTTTCTCGCCACGTTCGCCGGCTTTCCCCGTCAAGCTCTAAATCGGGGGCTCCCTTTAGGGTTCCGATTTAGTGCTTTACGGCACCTCGACCCCAAAAAACTTGATTAGGGTGATGGTTCACGTAGTGGGCCATCGCCCTGATAGACGGTTTTTCGCCCTTTGACGTTGGAGTCCACGTTCTTTAATAGTGGACTCTTGTTCCAAACTGGAACAACACTCAACCCTATCTCGGTCTATTCTTTTGATTTATAAGGGATTTTGCCGATTTCGGCCTATTGGTTAAAAAATGAGCTGATTTAACAAAAATTTAACGCGAATTTTAACAAAATATTAACGTTTACAATTTCAGGTGGCACTTTTCGGGGAAATGTGCGCGGAACCCCTATTTGTTTATTTTTCTAAATACATTCAAATATGTATCCGCTCATGAATTAATTCTTAGAAAAACTCATCGAGCATCAAATGAAACTGCAATTTATTCATATCAGGATTATCAATACCATATTTTTGAAAAAGCCGTTTCTGTAATGAAGGAGAAAACTCACCGAGGCAGTTCCATAGGATGGCAAGATCCTGGTATCGGTCTGCGATTCCGACTCGTCCAACATCAATACAACCTATTAATTTCCCCTCGTCAAAAATAAGGTTATCAAGTGAGAAATCACCATGAGTGACGACTGAATCCGGTGAGAATGGCAAAAGTTTATGCATTTCTTTCCAGACTTGTTCAACAGGCCAGCCATTACGCTCGTCATCAAAATCACTCGCATCAACCAAACCGTTATTCATTCGTGATTGCGCCTGAGCGAGACGAAATACGCGATCGCTGTTAAAAGGACAATTACAAACAGGAATCGAATGCAACCGGCGCAGGAACACTGCCAGCGCATCAACAATATTTTCACCTGAATCAGGATATTCTTCTAATACCTGGAATGCTGTTTTCCCGGGGATCGCAGTGGTGAGTAACCATGCATCATCAGGAGTACGGATAAAATGCTTGATGGTCGGAAGAGGCATAAATTCCGTCAGCCAGTTTAGTCTGACCATCTCATCTGTAACATCATTGGCAACGCTACCTTTGCCATGTTTCAGAAACAACTCTGGCGCATCGGGCTTCCCATACAATCGATAGATTGTCGCACCTGATTGCCCGACATTATCGCGAGCCCATTTATACCCATATAAATCAGCATCCATGTTGGAATTTAATCGCGGCCTAGAGCAAGACGTTTCCCGTTGAATATGGCTCATAACACCCCTTGTATTACTGTTTATGTAAGCAGACAGTTTTATTGTTCATGACCAAAATCCCTTAACGTGAGTTTTCGTTCCACTGAGCGTCAGACCCCGTAGAAAAGATCAAAGGATCTTCTTGAGATCCTTTTTTTCTGCGCGTAATCTGCTGCTTGCAAACAAAAAAACCACCGCTACCAGCGGTGGTTTGTTTGCCGGATCAAGAGCTACCAACTCTTTTTCCGAAGGTAACTGGCTTCAGCAGAGCGCAGATACCAAATACTGTCCTTCTAGTGTAGCCGTAGTTAGGCCACCACTTCAAGAACTCTGTAGCACCGCCTACATACCTCGCTCTGCTAATCCTGTTACCAGTGGCTGCTGCCAGTGGCGATAAGTCGTGTCTTACCGGGTTGGACTCAAGACGATAGTTACCGGATAAGGCGCAGCGGTCGGGCTGAACGGGGGGTTCGTGCACACAGCCCAGCTTGGAGCGAACGACCTACACCGAACTGAGATACCTACAGCGTGAGCTATGAGAAAGCGCCACGCTTCCCGAAGGGAGAAAGGCGGACAGGTATCCGGTAAGCGGCAGGGTCGGAACAGGAGAGCGCACGAGGGAGCTTCCAGGGGGAAACGCCTGGTATCTTTATAGTCCTGTCGGGTTTCGCCACCTCTGACTTGAGCGTCGATTTTTGTGATGCTCGTCAGGGGGGCGGAGCCTATGGAAAAACGCCAGCAACGCGGCCTTTTTACGGTTCCTGGCCTTTTGCTGGCCTTTTGCTCACATGTTCTTTCCTGCGTTATCCCCTGATTCTGTGGATAACCGTATTACCGCCTTTGAGTGAGCTGATACCGCTCGCCGCAGCCGAACGACCGAGCGCAGCGAGTCAGTGAGCGAGGAAGCGGAAGAGCGCCTGATGCGGTATTTTCTCCTTACGCATCTGTGCGGTATTTCACACCGCATATATGGTGCACTCTCAGTACAATCTGCTCTGATGCCGCATAGTTAAGCCAGTATACACTCCGCTATCGCTACGTGACTGGGTCATGGCTGCGCCCCGACACCCGCCAACACCCGCTGACGCGCCCTGACGGGCTTGTCTGCTCCCGGCATCCGCTTACAGACAAGCTGTGACCGTCTCCGGGAGCTGCATGTGTCAGAGGTTTTCACCGTCATCACCGAAACGCGCGAGGCAGCTGCGGTAAAGCTCATCAGCGTGGTCGTGAAGCGATTCACAGATGTCTGCCTGTTCATCCGCGTCCAGCTCGTTGAGTTTCTCCAGAAGCGTTAATGTCTGGCTTCTGATAAAGCGGGCCATGTTAAGGGCGGTTTTTTCCTGTTTGGTCACTGATGCCTCCGTGTAAGGGGGATTTCTGTTCATGGGGGTAATGATACCGATGAAACGAGAGAGGATGCTCACGATACGGGTTACTGATGATGAACATGCCCGGTTACTGGAACGTTGTGAGGGTAAACAACTGGCGGTATGGATGCGGCGGGACCAGAGAAAAATCACTCAGGGTCAATGCCAGCGCTTCGTTAATACAGATGTAGGTGTTCCACAGGGTAGCCAGCAGCATCCTGCGATGCAGATCCGGAACATAATGGTGCAGGGCGCTGACTTCCGCGTTTCCAGACTTTACGAAACACGGAAACCGAAGACCATTCATGTTGTTGCTCAGGTCGCAGACGTTTTGCAGCAGCAGTCGCTTCACGTTCGCTCGCGTATCGGTGATTCATTCTGCTAACCAGTAAGGCAACCCCGCCAGCCTAGCCGGGTCCTCAACGACAGGAGCACGATCATGCGCACCCGTGGGGCCGCCATGCCGGCGATAATGGCCTGCTTCTCGCCGAAACGTTTGGTGGCGGGACCAGTGACGAAGGCTTGAGCGAGGGCGTGCAAGATTCCGAATACCGCAAGCGACAGGCCGATCATCGTCGCGCTCCAGCGAAAGCGGTCCTCGCCGAAAATGACCCAGAGCGCTGCCGGCACCTGTCCTACGAGTTGCATGATAAAGAAGACAGTCATAAGTGCGGCGACGATAGTCATGCCCCGCGCCCACCGGAAGGAGCTGACTGGGTTGAAGGCTCTCAAGGGCATCGGTCGAGATCCCGGTGCCTAATGAGTGAGCTAACTTACATTAATTGCGTTGCGCTCACTGCCCGCTTTCCAGTCGGGAAACCTGTCGTGCCAGCTGCATTAATGAATCGGCCAACGCGCGGGGAGAGGCGGTTTGCGTATTGGGCGCCAGGGTGGTTTTTCTTTTCACCAGTGAGACGGGCAACAGCTGATTGCCCTTCACCGCCTGGCCCTGAGAGAGTTGCAGCAAGCGGTCCACGCTGGTTTGCCCCAGCAGGCGAAAATCCTGTTTGATGGTGGTTAACGGCGGGATATAACATGAGCTGTCTTCGGTATCGTCGTATCCCACTACCGAGATATCCGCACCAACGCGCAGCCCGGACTCGGTAATGGCGCGCATTGCGCCCAGCGCCATCTGATCGTTGGCAACCAGCATCGCAGTGGGAACGATGCCCTCATTCAGCATTTGCATGGTTTGTTGAAAACCGGACATGGCACTCCAGTCGCCTTCCCGTTCCGCTATCGGCTGAATTTGATTGCGAGTGAGATATTTATGCCAGCCAGCCAGACGCAGACGCGCCGAGACAGAACTTAATGGGCCCGCTAACAGCGCGATTTGCTGGTGACCCAATGCGACCAGATGCTCCACGCCCAGTCGCGTACCGTCTTCATGGGAGAAAATAATACTGTTGATGGGTGTCTGGTCAGAGACATCAAGAAATAACGCCGGAACATTAGTGCAGGCAGCTTCCACAGCAATGGCATCCTGGTCATCCAGCGGATAGTTAATGATCAGCCCACTGACGCGTTGCGCGAGAAGATTGTGCACCGCCGCTTTACAGGCTTCGACGCCGCTTCGTTCTACCATCGACACCACCACGCTGGCACCCAGTTGATCGGCGCGAGATTTAATCGCCGCGACAATTTGCGACGGCGCGTGCAGGGCCAGACTGGAGGTGGCAACGCCAATCAGCAACGACTGTTTGCCCGCCAGTTGTTGTGCCACGCGGTTGGGAATGTAATTCAGCTCCGCCATCGCCGCTTCCACTTTTTCCCGCGTTTTCGCAGAAACGTGGCTGGCCTGGTTCACCACGCGGGAAACGGTCTGATAAGAGACACCGGCATACTCTGCGACATCGTATAACGTTACTGGTTTCACATTCACCACCCTGAATTGACTCTCTTCCGGGCGCTATCATGCCATACCGCGAAAGGTTTTGCGCCATTCGATGGTGTCCGGGATCTCGACGCTCTCCCTTATGCGACTCCTGCATTAGGAAGCAGCCCAGTAGTAGGTTGAGGCCGTTGAGCACCGCCGCCGCAAGGAATGGTGCATGCAAGGAGATGGCGCCCAACAGTCCCCCGGCCACGGGGCCTGCCACCATACCCACGCCGAAACAAGCGCTCATGAGCCCGAAGTGGCGAGCCCGATCTTCCCCATCGGTGATGTCGGCGATATAGGCGCCAGCAACCGCACCTGTGGCGCCGGTGATGCCGGCCACGATGCGTCCGGCGTAGAGGATCGAGATCTCGATCCCGCGAAATTAATACGACTCACTATAGGGGAATTGTGAGCGGATAACAATTCCCCTCTAGAAATAATTTTGTTTAACTTTAAGAAGGAGATATACCATGGGCAGCAGCCATCATCATCATCATCACAGCAGCGGCCTGGTGCCGCGCGGCAGCCATGACTCAGAAGTCAATCAAGAAGCTAAGCCAGAGGTCAAGCCAGAAGTCAAGCCTGAGACTCACATCAATTTAAAGGTGTCCGATGGATCTTCAGAGATCTTCTTCAAGATCAAAAAGACCACTCCTTTAAGAAGGCTGATGGAAGCGTTCGCTAAAAGACAGGGTAAGGAAATGGACTCCTTAAGATTCTTGTACGACGGTATTAGAATTCAAGCTGATCAGGCCCCTGAAGATTTGGACATGGAGGATAACGATATTATTGAGGCTCACCGCGAACAGATTGGAGGTAGCAGCGAGAACCTGTACTTCCAGGGACATATGGGAATGAAAACACTGGAACGTAAAATCTACGGTCACGGTGTCTTAATGATCCTGAGTACCCTTATTTTTGGCCTCTTCCTCTGGATGAATCTCGTGGGTGGTTTCGAAATCGTACCTGGTTACATTATTAATTTTAACATTCCAGGTACAGCTGAAGGTTGGGCTAAAGCTCACGTTGGTCCTGCTTTAAACGGTATGATGGTTATTGCGATTGGTCTTGTATTGCCAAAATTAGCATTCCCGTTAAAAACTGCAAAAAAACTTGGTTACATTATCGTATTAGATGGTTGGGGTAATGTCTGTTTCTATTTCTTTAGCAACTTTGCTCCATCTCGTGGTCTATCTTTTGGCAGTAACCGTCTTGGCGAAACTAACATCTTTGGCGTACTTGCCCTGGCTCCGGCGTACGTGTTCGGTGTTTTAGCCATTGGTGCTTTGGCAATTATTGGTTTTAAGGCTCTTCGTCAAACTGATGCTGAAGATCGTGCTATTGAAAGTGCTAATGCATGAAAGCTTGCGGCCGCACTCGAGCACCACCACCACCACCACTGAGATCCGGCTGCTAACAAAGCCCGAAAGGAAGCTGAGTTGGCTGCTGCCACCGCTGAGCAATAACTAGCATAACCCCTTGGGGCCTCTAAACGGGTCTTGAGGGGTTTTTTGCTGAAAGGAGGAACTATATCCGGAT
